# Simultaneous determination of pigments of spinach (*Spinacia oleracea* L.) leaf for quality inspection using hyperspectral imaging and multi-task deep learning regression approaches

**DOI:** 10.1016/j.fochx.2024.101481

**Published:** 2024-05-17

**Authors:** Mengyu He, Chen Jin, Cheng Li, Zeyi Cai, Dongdong Peng, Xiang Huang, Jun Wang, Yuanning Zhai, Hengnian Qi, Chu Zhang

**Affiliations:** School of Information Engineering, Huzhou University, 313000 Huzhou, China

**Keywords:** Hyperspectral imaging, Spinach, Pigments, Multi-task regression, Convolutional neural networks

## Abstract

Rapid and accurate determination of pigment content is important for quality inspection of spinach leaves during storage. This study aimed to use hyperspectral imaging at two spectral ranges (visible/near-infrared, VNIR: 400–1000 nm; NIR: 900–1700 nm) to simultaneously determine the pigment (chlorophyll *a*, chlorophyll *b*, total chlorophyll, and carotenoids) content in spinach stored at different durations and conditions (unpackaged and packaged). Partial least squares (PLS), back propagation neural network (BPNN) and convolutional neural network (CNN) were used to establish single-task and multi-task regression models. Single-task CNN (STCNN) models and multi-task CNN (MTCNN) models obtained better performances than the other models. The models using VNIR spectra were superior to those using NIR spectra. The overall results indicated that hyperspectral imaging with multi-task learning could predict the quality attributes of spinach simultaneously for spinach quality inspection under various storage conditions. This research will guide food quality inspection by simultaneously inspecting multiple quality attributes.

## Highlights

• Spinach pigments content was measured using hyperspectral imaging.
• Single-task and multi-task models were built using machine and deep learning models.
• Single-task models performed slightly better than the corresponding multi-task models.
• Multi-task models showed advantage of simultaneously predicting multiple attributes.
• The important wavelengths of deep learning models were visualized and identified.

## 1 Introduction

Vegetables are common foods in daily life. The quality of vegetables is the focus of a healthy diet, and freshness is an important factor affecting vegetable quality. To extend the shelf-life and maintain the freshness of vegetables, methods such as refrigeration and wrapping with cling film (packaging) to prevent water loss and spoilage are used. With the extension of storage time, the internal components of vegetables will undergo certain changes (Salehi, 2020; Siripongvutikorn, Usawakesmanee, Pisuchpen, Khatcharin, & Rujirapong, 2023; Spinardi, Cocetta, Baldassarre, Ferrante, & Mignani, 2010). Subtle changes within the vegetables during storage are difficult to be identified with the naked eye, affecting the evaluation of the vegetable quality and freshness.

Sensory methods to detect the appearance, texture and smell can be used to evaluate the quality of vegetables. Although sensory analysis methods are widely adopted, they require trained and experienced experts, which cannot be used for large scale monitoring. During the storage of vegetables, the measurement of quality attributes helps the assessment of the true quality and freshness of the vegetables. Many scholars have studied the variation of quality attributes in vegetables using different chemical methods (Kevers et al., 2007; Kumar, 2017; Limantara, Dettling, Indrawati, Indriatmoko, & Brotosudarmo, 2015). There are also drawbacks to these methods, such as time-consuming, labor-intensive, complex, reagent waste, and the destruction of experimental samples, etc. Therefore, to quickly and accurately evaluate the quality and freshness of vegetables, it is of great significance to seek a fast and non-destructive detection method.

Hyperspectral imaging is a non-destructive analytical technology that combines spectroscopy and imaging technology to obtain spatial and spectral information simultaneously (Gowen, O'Donnell, Cullen, Downey, & Frias, 2007; Siche et al., 2016; Wieme et al., 2022). Many scholars have used hyperspectral techniques to detect food freshness during storage (Chen, Wang, Zhang, & Nie, 2021; Zhu, Feng, Zhang, Bao, & He, 2019). Some scholars have also utilized hyperspectral imaging and combined different data analysis methods to study the quality attributes of food (Meghar et al., 2023; Song et al., 2023; Zhang et al., 2020). There are plenty of quality attributes of food. Generally, one regression model is built for only one quality attribute, which is a single-task issue. To predict multiple quality attributes, the corresponding number of regression models are built for each quality attribute separately. By applying these models, the quality attributes can be predicted one by one to achieve the multiple prediction (Duan, Zhu, Yao, & Lewis, 2017; He, Zhang, Zhou, & He, 2021; Squeo et al., 2022). Different regression models can be built using the same spectra and different quality attributes. Although simultaneous prediction has been mentioned in some studies, the final results were still modeled with a single-task for each quality attribute (Cheng et al., 2016; He et al., 2021; Li et al., 2021). Some common single-task regression models for various food quality studies are partial least square regression (PLSR), support vector regression (SVR), multiple linear regression (MLR), back-propagation neural networks (BPNN), least-square support vector machine (LS-SVM), etc. (Liu et al., 2021; Wang, Lin, Xu, Bi, & Sun, 2021; Zhuang et al., 2022). From the above research, it can be seen that the evaluation of food quality is mostly achieved by establishing a single-task model. However, for practical applications in food engineering, multiple quality attributes predictions are always required. Based on those mentioned conventional single-task learning methods, several different models should be trained, optimized and loaded individually to predict the corresponding quality attribute one by one. This approach has low efficiency. It is necessary to explore the approaches to predict the multiple quality attributes using only one model instead of multiple models.

Multitask learning (MTL) is to learn multiple related tasks together. Useful information contained in multiple learning tasks is utilized to assist each learning task with facilitating more efficient processing of the original subtasks (Qin et al., 2022). Multi-task learning has been successfully applied to natural language processing (Worsham & Kalita, 2020), computer vision (Huang et al., 2019) and other fields. Conventional multi-task learning such as PLSR (Mishra, Verschoor, Polder, & Boer, 2023), BPNN and other algorithms (Liu, Wang, Tan, Ma, & Xu, 2020) are used for multi-task regression issues. Deep learning-based MTL, such as multi-task convolutional neural network (Mishra & Passos, 2022), has gained more and more attention due to the strong feature learning and representation ability of deep learning. Deep learning algorithms can mine the deep information from the data, while deep learning-based multi-task learning can obtain more integrated and variable information.

**Fig. 1:**
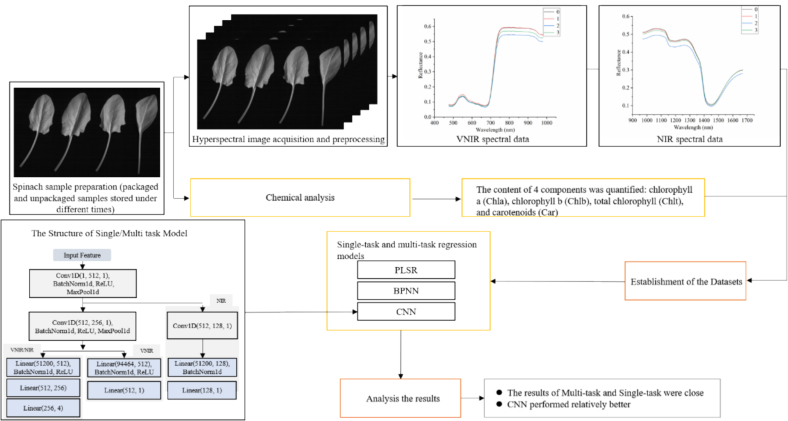
The overall analysis procedure of this study.

Color is one of the main attributes relating to the sensory quality of spinach. The color mainly reflects the freshness of spinach and the color of spinach changes along with the storage and processing (Kidmose, Edelenbos, Christensen, & Hegelund, 2005). This study combined hyperspectral imaging with multi-task deep learning to investigate the simultaneous determination of pigments (chlorophyll *a* (Chla), chlorophyll *b* (Chlb), total chlorophyll (Chlt), and carotenoids (Car)) content of spinach during storage under the unpackaged and packaged (using cling film) situation. The specific objectives were: 1) to establish the single-task regression models using PLSR, BPNN and CNN to predict the content of Chla, Chlb, Chlt and Car in spinach leaves during storage under the unpackaged and packaged situation; 2) to establish the multi-task regression models using PLSR, BPNN and CNN to predict the content of Chla, Chlb, Chlt and Car in spinach leaves during storage under the unpackaged and packaged situation; 3) to compare the performance of single-task and multi-task models to explore a fast and nondestructive method for assessing the quality of spinach leaves during storage under the unpackaged and packaged situation; 4) to visualize and compare the important wavelengths of the single-task CNN and multi-task CNN for the pigments content prediction. The overall analysis procedure of this study is shown in Fig. 1.

## 2 Materials and methods

### 2.1 Sample preparation

The spinach used in the experiment was purchased from a local market in Huzhou, Zhejiang Province, China. The ready-to-sell mature spinach samples were sent to the laboratory immediately after the harvest from the field in the early morning of 11th and 12th April 2023. To minimize the uncertain factors in the experiment, the leaves were cleaned and dried with tissues, and then stored at room temperature. The reason for using packaged and unpackaged spinach was to explore the feasibility of the multi-task learning approaches with hyperspectral imaging under different storage conditions.

For the unpackaged spinach leaves, the cleaned leaves were stored for four storage periods (0 h, 3 h, 6 h, 9 h), and then used for hyperspectral image acquisition and pigment contents measurement. The storage periods were determined by the pre-experiments, and some differences could be observed during these storage periods. The hyperspectral images of unpackaged spinach leaves were collected in two days. On the first day, 60 spinach samples were sorted at room temperature (20 ± 2 °C). Then, 15 spinach leaves were randomly selected at each period to acquire hyperspectral images using the two cameras at different spectral ranges. The experimental process was then repeated on the second day at the same period with 60 new spinach samples. Ultimately, hyperspectral images of 120 samples were obtained for each camera.

For the packaged spinach leaves, every cleaned leaf was packaged with the cling film, and stored at room temperature (23 ± 2 °C). The experiment was carried out in five storage periods (0 h, 3 h, 6 h, 9 h, 24 h). For each period, 30 spinach samples were randomly selected at different periods and hyperspectral images of two cameras at different spectral ranges were acquired. Thus, 150 packaged samples were used for analysis. The leaves were all different, and no duplicate samples were used in different storage periods. The storage time used in this research was determined by pre-experiments to explore the time of treatments.

### 2.2 Hyperspectral image acquisition and spectra extraction

#### 2.2.1 HSI system and image calibration

The hyperspectral imaging system used in the experiment was a laboratory-based equipment setup. The imaging system consists of two cameras, a LabScanner platform (Spectral Imaging Ltd., Oulu, Finland) which integrates of a camera holder, a linear light source with six halogen lamps (OSRAM, Munich, Germany) and a mobile platform, and the corresponding control software (LUMO-Scanner, Spectral Imaging Ltd., Oulu, Finland). During the experiment, the camera head types were FX10 (spectral range: 400–1000 nm) (Spectral Imaging Ltd., Oulu, Finland) and FX17 (spectral range: 900–1700 nm) (Spectral Imaging Ltd., Oulu, Finland), and the distance between the lens and the moving platform was 300 mm. During the scanning, the hyperspectral lens was fixed, and the sample is moved through the integrated mobile platform. The movement speed of the samples for the FX10 camera was 34 mm/s, and the movement speed of the samples for the FX17 camera platform was 26.3 mm/s. To acquire hyperspectral images, three or four leaves were placed on the moving platform each time, and they were disconnected from each other.

The obtained raw hyperspectral images should be corrected by removing the dark current and calibrating the light intensity. Thus, it was necessary to perform black and white correction of the hyperspectral image according to eq. (1):

(1) $$I=\frac{S-B}{W-B}$$

where *S* is the original hyperspectral image of spinach leaves, *B* is the dark reference image with a reflectance close to 0%, and *W* is the white reference image with a reflectance close to 100%.

#### 2.2.2 Spectra extraction and preprocessing

After the acquisition and correction of the hyperspectral images, the spectral information of the samples in the hyperspectral images was extracted after a series of processing. Firstly, the hyperspectral image acquisition system automatically generated RGB images and hyperspectral images with the same spatial dimensions during image acquisition. Second, a mask was constructed using binarization to separate the leaf from the background in the RGB image, and the mask was applied to the corresponding hyperspectral image to isolate the samples from the background. The whole leaf area was defined as the ROI. The average spectrum of ROI was extracted to represent the whole sample. To eliminate the impact of noise generated by the camera itself and the external environment on the spectrum, the head and tail of the original spectrum were cut off for subsequent data analysis and model construction. For the FX10 and FX17 cameras, the wavelengths were retained in the range of 475–980 nm (VNIR) and 970–1670 nm (NIR), respectively. No spectral preprocessing methods were applied to the spectral data.

### 2.3 Measurement of pigments in spinach leaves

#### 2.3.1 Ultraviolet spectrophotometer (UV–vis)

In this study, the spectrophotometric method was utilized to determine the content of chlorophyll in spinach leaves. The ultraviolet-visible spectrophotometer system (L5S, INESA Analytical Instrument Co. Ltd., Shanghai, Chian) adopts advanced optical, mechanical, and electrical designs with stable performance. The wavelength is input through the LCD touch display, which makes it flexible and convenient to operate. The light source adopts an imported 12 V, 20 W halogen tungsten lamp, with a wavelength range of 325–1100 nm.

#### 2.3.2 Extraction and measurement of pigments

To extract pigments, about 0.1 g of leaf sample was cut, weighed and put into a 10 mL test tube, and 10 mL of 95% alcohol (Shanghai Lingfeng Chemical Reagent Co. LTD., Shanghai, China) was injected into the test tube using a pipette (Zhang, Wang, Liu, He, & Xiao, 2017). A dark environment was required throughout the entire pigment extraction process. After all the samples were processed, the alcohol-filled tubes were placed at room temperature and dark environment, and the pigment measurements were performed after 48 h of extraction.

To measure the content of the pigments, 95% alcohol was selected as a control for the sample group. The extract in the tube was shaken well and poured into a cuvette, and the cuvette was put into the UV spectrophotometer sequentially. Six cuvettes could be measured each time; three bands of 665 nm, 649 nm, and 470 nm were selected for absorbance measurement through the control screen. After the experimental data were obtained, the concentrations of chlorophyll *a* (Chla), chlorophyll *b* (Chlb), total chlorophyll (Chlt) and carotenoids (Car) were calculated using the same way as the research of Zhang et al. (2017).

### 2.4 Outlier removal and dataset split

This study investigated the changes in freshness of spinach in both packaged and unpackaged forms at different periods. To further develop the regression models, the outlier in the data needs to be removed first for better prediction results. The predicted values of each pigment attribute of all samples were obtained by the single-task partial least squares regression (PLSR) model, and the absolute values of the difference between the true and predicted values were calculated. The outlier thresholds were manually defined for each pigment attribute (Zhang et al., 2023). As the multi-task regression model predicted the pigment attributes simultaneously, a sample value was judged to be an outlier under any one of the metrics, and the other three metrics also removed it as an outlier. The number of outliers removed varied for the two cameras.

After removal, the dataset was randomly divided into the training, validation and testing set according to the ratio of 3:1:1. The division of the different datasets and the statistical summary of the different pigment indicators are shown in **Table S1** and **Table S2** (in **Appendices**) for the two spectral ranges, respectively. It should be noted that the above-mentioned dataset split was conducted for multi-task regression, and the datasets used for single-task regression were the same as those for multi-task regression.

### 2.5 Data analysis and visualization

#### 2.5.1 Data analysis methods

##### 2.5.1.1 Partial least squares regression

Partial least squares regression (PLSR) is a multivariate statistical data analysis method (Geladi & Kowalski, 1986), which is based on the principle of finding a linear regression model by projecting the predictor and observed variables into a new space. It is an analytical method that combines the advantages of principal component analysis, typical correlation analysis, and multiple linear regression analysis; Compared with principal component analysis (Abdi & Williams, 2010), the PLS method has a “response” matrix, so it has a predictive function. In this study, a grid search method was used to determine the best principal component score for each model. Generally, the PLS for single-task is treated as PLS1 (generally shortened as PLS), and the PLS for multi-task is treated as PLS2 (Kelley, Rials, Snell, Groom, & Sluiter, 2004).

##### 2.5.1.2 Back propagation neural network

Back propagation neural network (BPNN) is a multilayer feedforward neural network trained according to the error backpropagation algorithm (Li, Cheng, Shi, & Huang, 2012). The common BPNN consists of three layers: the input layer, the hidden layer, and the output layer. The process of data propagation in the network is as follows: Firstly, the data is passed in through the input layer, and then the weights and thresholds are adjusted and calculated in the hidden layer and output layer, respectively, to realize the nonlinear transformation. Finally, the predicted value is output through the output layer, and the error is obtained by comparing it with the target value. BPNN can also be used for single-task and multi-task learning (Liu et al., 2020).

##### 2.5.1.3 Single-task convolutional neural networks

Convolutional Neural Networks (CNN) is one of the most common deep learning algorithms (Malek, Melgani, & Bazi, 2018). It is a type of feedforward neural network that includes convolutional computation and it also has a deep learning structure, which can perform supervised and unsupervised learning. The most common CNN structures include input layer, hidden layer, and output layer, where hidden layers include convolutional layer, batch normalization layer (BN), activation layer (ReLU), pooling layer (Pooling), and fully connected layer (Linear). The biggest advantage is that it shares convolutional kernels and can process high-dimensional data, and it does not require manual feature selection. This study adopted a STCNN architecture as shown in **Fig. S1**.

**Fig. S1** (a) shows the structure of the STCNN network based on VNIR spectra. The same network structure diagrams were created for the four attributes of unpackaged and packaged spinach leaves. From the figure, it could be seen that the network structure was composed of two convolutional blocks and two fully connected layers. Each convolutional block includes a convolutional layer, a batch normalization layer, an activation layer, and a pooling layer. Firstly, the data features were first input, after which the parameters and weights were adjusted by a series of calculations, and the predicted value of an attribute was output through the fully connected layer.

**Fig. S1** (b) shows the structure of the STCNN network based on NIR spectra. Except for the network structure of Car in packaged spinach leaves, which differed from that shown in **Fig. S1** (b), the same network structure diagrams were created for the four attributes in other unpackaged and packaged spinach leaves. It could be seen that the structure was similar to the STCNN structure based on VNIR spectra. The difference was that there was only one convolutional layer in the second convolutional block of the network. By inputting data and undergoing a series of transformation calculations, the predicted value of an attribute was output through the fully connected layer. The difference in the network structure for packing Car of spinach leaves was that there was only one convolutional block because better results could be obtained when using one convolutional block.

##### 2.5.1.4 Multi-task convolutional neural networks

Multi-task learning (MTL) is the process of learning multiple related tasks together, sharing some parameters between multiple tasks, and sharing the learned information during the learning process (Thung & Wee, 2018). It has better generalization ability compared with single-task learning. The mode of MTL is divided into a hard sharing mechanism for parameters and soft sharing mechanism for parameters. This study adopted a hard sharing mechanism of MTL parameters, which shared hidden layers among all tasks while retaining several task-specific output layers.

Multi-task convolutional neural network (MTCNN) is a deep learning method that combines multi-task learning (MTL) and convolutional neural networks (CNN) (Li et al., 2022). The CNN was used as a shared layer for multi-task learning, four tasks were learned simultaneously, and the predicted values of the four tasks were output. In the network, the loss function used was the MSE Loss, whose return value was the average of the loss sum. The network was trained using the following method for assigning weights: first, the variance of the actual values of each task was found, and then the variance was multiplied by the weights to equal 0.25, and finally the weights were calculated. It was worth noting that the weights for each attribute were assigned using this approach. The purpose of this method was to reduce the weight of tasks with larger contributions and increase the weight of tasks with smaller contributions, thereby improving the predictive performance of multi-task models for each task. This study uses the same MTCNN model based on VNIR and NIR, and its architecture is shown in **Fig. S2**. It could be seen that the structure of the MTCNN network was similar to the STCNN structure, both mainly composed of two convolutional blocks and two fully connected layers. The biggest difference was that the last fully connected layer outputs the predicted values of four attributes simultaneously.

In addition to the above-mentioned network model structures, single-task partial least squares regression (STPLSR), single-task backpropagation neural network (STBPNN) and multi-task partial least squares regression (MTPLSR), multi-task backpropagation neural network (MTBPNN) model structures were compared.

#### 2.5.2 Visualization of CNN models using grad-CAM++

Gradient-weighted Class Activation Mapping++ (Grad-CAM++) is a visual convolutional neural network map based on CAM and Grad-CAM that provides good visual interpretability of neural network models (Chattopadhay, Sarkar, Howlader, & Balasubramanian, 2018). First, the data and categories of interest are entered, and the gradient is set to zero for all categories except the category of interest. Secondly, the category that the predicted data belongs to is obtained through forward propagation and the location of the feature is obtained using back propagation. The calculation of weights in Grad-CAM++ is refined to account for the impact of each feature by incorporating the second-order derivatives of the gradients, highlighting the contribution of each feature towards the output with higher accuracy. This nuanced approach to weight calculation helps identify which features most strongly influence the predictions of models. Finally, the global average pooling layer (GAP) is performed on the gradient feature map to obtain the desired weights. The gradient-weighted class activation map is obtained by multiplying each feature with the corresponding weight. The weights derived from the global average pooling layer represent the importance of each feature in the activation maps for contributing to the output of models for the specific task. These weights are crucial as they highlight which features in the input are most influential in the predictions of models.

In this study, the multi-task regression model was trained based on the hard parameter-sharing approach so that the four tasks had the same model parameters. Grad-CAM++ was utilized to visualize the regression model for this study. Important wavelengths were visualized for different single-task regression models as well as for multi-task regression models. The visualization provided by one-dimensional Grad-CAM++ generates weight maps that indicate the significance of each wavelength in predicting the target variable. The intensity of weight values in the weight maps correlates directly with the importance of the wavelengths, where higher values signify higher importance. This method allows for a detailed inspection of how different wavelengths contribute to the predictions, and can reveal if the model is relying on sensible input features, thereby assessing the reliability and interpretability of the model. Additionally, the distributions of important wavelengths for single-task and multi-task models were compared. By interpreting the one-dimensional Grad-CAM++ visualizations, it is possible to discern not only the impact of specific wavelengths on the predicted outcomes but also how these impacts vary across different tasks within the multi-task model. This insight is crucial for refining the performance of models and understanding cross-task interactions.

### 2.6 Model performance evaluation and software

In the above model performance evaluation, the correlation coefficient (*r*) of the training set (*r*_*c*_), the validation set (*r*_*v*_), and the testing set (*r*_*p*_), and the root mean square error of the training set (RMSEC), the validation set (RMSEV), and the testing set (RMSEP) were used as attributes to evaluate the model performance. The closer the *r* is to 1, while the root mean square error is close to 0, the better the model performance is.

The STPLSR and MTPLSR models were implemented on PyCharm Community Edition (version 2021.2.1) (JetBrains, Prague, Czech Republic) with Python (version 3.10.8) and Scikit Learn (version 1.1.2). STCNN, STBPNN, MTCNN, MTBPNN, MTCNN and Grad-CAM++ were executed on the deep learning framework Pytorch (version 1.13.0). All data analysis was implemented on a computer with 11th Gen Intel (R) Core (TM) i7-11700K, 2.50 GHz, and 32GB RAM, with a GPU of GeForce RTX 3060.

## 3 Results and discussion

### 3.1 Results of pigment content regression models

#### 3.1.1 Results of single-task regression models

In this study, three single-task regression models, STPLSR, STBPNN, and STCNN were established using the divided datasets. The prediction results of these models for unpackaged and packaged spinach leaves are shown in Table 1 and Table 2.

**Table 1:** Prediction results of Chla, Chlb, Chlt, and Car of unpackaged spinach leaves by three regression models STPLSR, STBPNN, and STCNN, respectively. (Chla represents the chlorophyll *a*, Chlb represents the chlorophyll *b*, Chlt represents the total chlorophyll, and Car represents the carotenoids.)

Spectral range	Attributes	Model	*r*_*c*_	RMSEC (mg/g)	*r*_*v*_	RMSEV (mg/g)	*r*_*p*_	RMSEP (mg/g)
VNIR	Chla	STPLSR	0.9061	0.1328	0.5169	0.3357	0.7502	0.2251
		STBPNN	0.8625	0.1790	0.8263	0.2321	0.8614	0.1825
		STCNN	0.9470	0.1157	0.8039	0.2443	0.8036	0.1933
	Chlb	STPLSR	0.9967	0.0165	0.6421	0.1831	0.5566	0.1768
		STBPNN	0.8312	0.2374	0.8149	0.2234	0.8127	0.2712
		STCNN	**0.9632**	**0.0673**	**0.8527**	**0.1296**	**0.8529**	**0.1145**
	Chlt	STPLSR	0.9108	0.2067	0.5659	0.5367	0.7057	0.3742
		STBPNN	0.8112	0.4054	0.8026	0.5750	0.8002	0.3163
		STCNN	**0.9034**	**0.1194**	**0.8053**	**0.1560**	**0.8075**	**0.1786**
	Car	STPLSR	0.7057	0.0345	0.5392	0.0454	0.6984	0.0422
		STBPNN	0.7637	0.2375	0.7110	0.2482	0.7004	0.2271
		STCNN	0.8109	0.0904	0.6185	0.0890	0.6601	0.1014
NIR	Chla	STPLSR	0.9642	0.1009	0.7979	0.2266	0.7704	0.1941
		STBPNN	0.8251	0.8157	0.7288	0.8691	0.7227	0.7576
		STCNN	**0.8670**	**0.2686**	**0.7525**	**0.3319**	**0.7992**	**0.2985**
	Chlb	STPLSR	0.9651	0.0657	0.8022	0.1296	0.7296	0.1356
		STBPNN	0.7088	0.3262	0.6894	0.4173	0.7098	0.3314
		STCNN	**0.9144**	**0.1350**	**0.8514**	**0.1994**	**0.8859**	**0.2274**
	Chlt	STPLSR	0.9665	0.1592	0.8050	0.3484	0.7642	0.3158
		STBPNN	0.7644	0.4026	0.7407	0.4404	0.8040	0.2921
		STCNN	0.8612	1.6561	0.7486	1.6907	0.7661	1.6970
	Car	STPLSR	0.4624	0.0454	0.4882	0.0435	0.7095	0.0344
		STBPNN	0.7083	0.0733	0.6472	0.0703	0.6935	0.0763
		STCNN	0.8089	0.0339	0.7523	0.0348	0.7543	0.0351

**Table 2:** Prediction results of chlorophyll *a*, chlorophyll *b*, total chlorophyll, and carotenoids of packaged spinach leaves by three regression models STPLSR, STBPNN, and STCNN, respectively. (Chla represents the chlorophyll *a*, Chlb represents the chlorophyll *b*, Chlt represents the total chlorophyll, and Car represents the carotenoids.)

Spectral range	Attributes	Model	*r*_*c*_	RMSEC (mg/g)	*r*_*v*_	RMSEV (mg/g)	*r*_*p*_	RMSEP (mg/g)
VNIR	Chla	STPLSR	**0.9868**	**0.0956**	**0.9439**	**0.1962**	**0.9670**	**0.1749**
		STBPNN	**0.9689**	**0.1747**	**0.9616**	**0.1827**	**0.9628**	**0.1733**
		STCNN	0.9575	0.1722	0.9093	0.2292	0.9064	0.2556
	Chlb	STPLSR	0.9809	0.0754	0.9021	0.1621	0.9409	0.1569
		STBPNN	0.9587	0.3194	0.9358	0.3229	0.9363	0.2679
		STCNN	0.9389	0.1541	0.8791	0.1742	0.8515	0.2247
	Chlt	STPLSR	**0.9853**	**0.1664**	**0.9343**	**0.3465**	**0.9642**	**0.3030**
		STBPNN	**0.9699**	**0.4462**	**0.9576**	**0.4516**	**0.9581**	**0.3879**
		STCNN	0.9697	0.2586	0.9007	0.3869	0.9001	0.4606
	Car	STPLSR	0.6531	0.0572	0.7206	0.0580	0.5682	0.0599
		STBPNN	0.8187	0.0807	0.8436	0.0802	0.8225	0.0738
		STCNN	0.7760	0.0921	0.6754	0.0904	0.6892	0.0943
NIR	Chla	STPLSR	**0.9163**	**0.2196**	**0.8870**	**0.2930**	**0.8813**	**0.3032**
		STBPNN	0.9198	1.0669	0.9166	0.9345	0.8674	1.0862
		STCNN	0.9080	0.8832	0.7978	0.8623	0.8168	0.9695
	Chlb	STPLSR	0.9144	0.1350	0.8514	0.1994	0.8859	0.2274
		STBPNN	0.9517	0.2567	0.8670	0.2729	0.8734	0.3987
		STCNN	**0.9114**	**0.6729**	**0.8124**	**0.6647**	**0.8646**	**0.7708**
	Chlt	STPLSR	0.9182	0.3482	0.8797	0.4797	0.8825	0.5280
		STBPNN	0.9000	0.7370	0.8152	1.0903	0.8259	0.9453
		STCNN	**0.9473**	**0.7850**	**0.8331**	**0.8254**	**0.8391**	**1.0390**
	Car	STPLSR	0.8478	0.0383	0.7619	0.0565	0.6996	0.0532
		STBPNN	0.8759	0.0600	0.7385	0.0858	0.7316	0.0882
		STCNN	0.8046	0.3221	0.7476	0.2900	0.6355	0.3016

As can be seen in Table 2, Chla, Chlb and Chlt were better predicted compared to Car in separated regression models based on VNIR spectra for unpacked spinach leaves. The STCNN model performed better than the STBPNN model and PLSR model. Among them, the optimal performance for Chla and Chlt prediction could reach more than 0.8. The model prediction results for Chla, Chlb and Chlt were relatively good in the separate pigment regression models based on NIR spectra. The STCNN model outperformed the STBPNN model and PLSR model in predicting Chlb with a *r*_*p*_ of 0.8859. An overall comparison revealed that *r* obtained through modeling based on the two spectra had similar trends, but the performance of the model built based on VNIR spectra was better than the corresponding model built based on NIR spectra. This could indicate that the wavelength range of VNIR spectra was more suitable for conducting spinach freshness studies.

As can be seen in Table 3, the predicted results of the three regression models were consistent with the trend of the results of the corresponding models in Table 2 for the Chla, Chlb, Chlt and Car. The *r* was more satisfactory for the predicted Chla, Chlb and Chlt by the three models. The overall results illustrated the relatively optimal performance of the STCNN model built based on VNIR spectra.

**Table 3:** Simultaneous prediction outputs of three regression models MTPLSR, MTBPNN and MTCNN for Chla, Chlb, Chlt and Car in unpackaged spinach leaves. (Chla represents the chlorophyll *a*, Chlb represents the chlorophyll *b*, Chlt represents the total chlorophyll, and Car represents the carotenoids.)

Spectral range	Attributes	Model	*r*_*c*_	RMSEC (mg/g)	*r*_*v*_	RMSEV (mg/g)	*r*_*p*_	RMSEP (mg/g)
VNIR	Chla	MTPLSR	0.8804	0.1488	0.8509	0.3157	0.8247	0.3061
		MTBPNN	0.9000	1.7551	0.8055	1.7215	0.8387	1.8408
		MTCNN	**0.8040**	**0.1684**	**0.8683**	**0.2034**	**0.8394**	**0.2145**
	Chlb	MTPLSR	0.8663	0.1012	0.7904	0.2268	0.7982	0.2079
		MTBPNN	0.8668	0.8702	0.8353	0.8493	0.7269	0.9247
		MTCNN	**0.8966**	**0.1573**	**0.8416**	**0.1306**	**0.8077**	**0.2190**
	Chlt	MTPLSR	0.8866	0.2316	0.8412	0.5262	0.8378	0.4954
		MTBPNN	0.8620	3.0452	0.8446	3.1034	0.8251	2.9118
		MTCNN	**0.9044**	**0.2913**	**0.8681**	**0.3064**	**0.8468**	**0.3882**
	Car	MTPLSR	0.7624	0.0315	0.7611	0.0472	0.6824	0.0489
		MTBPNN	0.8685	0.3110	0.7385	0.3188	0.7326	0.2971
		MTCNN	0.8924	0.0319	0.8133	0.0397	0.7485	0.0356
NIR	Chla	MTPLSR	0.9534	0.1149	0.8046	0.2268	0.8038	0.1967
		MTBPNN	0.7085	0.4807	0.7071	0.4678	0.7996	0.4463
		MTCNN	**0.8610**	**0.2369**	**0.7633**	**0.2732**	**0.8054**	**0.2237**
	Chlb	MTPLSR	0.9504	0.0781	0.8123	0.1291	0.8402	0.1151
		MTBPNN	0.6498	0.2012	0.6253	0.1869	0.7006	0.1599
		MTCNN	0.8007	0.4136	0.6822	0.4626	0.6821	0.4013
	Chlt	MTPLSR	0.9637	0.1655	0.8188	0.3426	0.8371	0.2914
		MTBPNN	0.7094	0.6367	0.6783	0.6407	0.7679	0.5720
		MTCNN	0.8447	0.5523	0.7385	0.6081	0.7338	0.5387
	Car	MTPLSR	0.7512	0.0338	0.7375	0.0360	0.7126	0.0339
		MTBPNN	0.6205	0.0519	0.6658	0.0543	0.8186	0.0443
		MTCNN	0.7644	0.0414	0.8006	0.0358	0.7441	0.0386

#### 3.1.2 Results of multi-task regression models

In addition to the single-task regression models, the corresponding multi-task regression models MTPLSR, MTBPNN, and MTCNN have also been established. The results are shown in Table 3 and Table 4. Multi-task regression models output multiple prediction results simultaneously.

**Table 4:** Simultaneous prediction outputs of three regression models MTPLSR, MTBPNN and MTCNN for Chla, Chlb, Chlt and Car in packaged spinach leaves. (Chla represents the chlorophyll *a*, Chlb represents the chlorophyll *b*, Chlt represents the total chlorophyll, and Car represents the carotenoids.)

Spectral range	Attributes	Model	*r*_*c*_	RMSEC (mg/g)	*r*_*v*_	RMSEV (mg/g)	*r*_*p*_	RMSEP (mg/g)
VNIR	Chla	MTPLSR	**0.9816**	**0.1127**	**0.9452**	**0.2852**	**0.9628**	**0.2115**
		MTBPNN	0.9643	0.4305	0.9535	0.4290	0.9347	0.4713
		MTCNN	0.9796	0.1828	0.9438	0.2647	0.9171	0.2607
	Chlb	MTPLSR	0.9713	0.0923	0.9221	0.2062	0.9549	0.1425
		MTBPNN	0.9648	0.2424	0.9198	0.2618	0.9096	0.3090
		MTCNN	0.9759	0.1409	0.9026	0.2275	0.8849	0.2086
	Chlt	MTPLSR	0.9809	0.1895	0.9396	0.4856	0.9647	0.3383
		MTBPNN	0.9686	0.4871	0.9450	0.5166	0.9309	0.5985
		MTCNN	**0.9799**	**0.2858**	**0.9278**	**0.4584**	**0.9086**	**0.4405**
	Car	MTPLSR	0.8276	0.0424	0.8644	0.0426	0.8206	0.0448
		MTBPNN	0.8373	3.5627	0.8584	3.5521	0.8036	3.5746
		MTCNN	0.9499	0.0291	0.8611	0.0482	0.7143	0.0551
NIR	Chla	MTPLSR	0.9143	0.2220	0.8874	0.3932	0.8453	0.3245
		MTBPNN	0.8914	0.7348	0.6665	0.8096	0.8129	0.8259
		MTCNN	0.8499	0.6277	0.6210	0.8446	0.7784	0.7291
	Chlb	MTPLSR	0.9170	0.1330	0.8460	0.2563	0.8413	0.2343
		MTBPNN	0.8886	0.4300	0.6914	0.4455	0.8582	0.5393
		MTCNN	0.8453	0.3345	0.6056	0.4774	0.7896	0.3773
	Chlt	MTPLSR	0.9181	0.3484	0.8763	0.6421	0.8481	0.5442
		MTBPNN	0.8990	1.5157	0.6686	1.5659	0.8295	1.6965
		MTCNN	0.8451	0.7972	0.6208	1.1674	0.7829	0.9395
	Car	MTPLSR	0.8016	0.0432	0.8167	0.0552	0.8136	0.0452
		MTBPNN	0.8141	0.3278	0.6052	0.3462	0.6045	0.3499
		MTCNN	0.8079	0.0935	0.6056	0.1185	0.7094	0.1195

As can be seen in Table 3, the prediction results were similar for the testing sets of Chla, Chlb and Chlt in the multitask modeling of unpacked spinach leaves based on VNIR spectra, with *r*_*p*_ above 0.8. Compared to the MTPLSR model and the MTBPNN model, the MTCNN model showed optimal performance for the *r* of the pigment indicators. Multi-task modeling based on NIR spectra showed similar prediction results to those based on VNIR spectra. Among them, the MTCNN model had better prediction results for Chla, with a *r*_*p*_ of 0.8054 for the testing set. By comparing the results of modeling based on the two spectra, it could be seen that the trend of *r* was similar but the results based on VNIR spectra were better than those based on NIR spectra. This again demonstrated that the range of VNIR spectra wavelengths was more suitable for spinach freshness studies than the range of NIR spectra wavelengths.

It can be seen from Table 4 that the models built based on both spectra gave better prediction results for Chla, Chlb and Chlt than for Car, which was consistent with the trend of the results in Table 3. The overall *r* in Table 4 was higher than that in Table 3. Overall, the performance of the MTCNN model built based on VNIR spectra was relatively optimal.

#### 3.1.3 Model performances comparison of single-task and multi-task models

The prediction results of the single-task and multi-task models are shown in Table 1, Table 2, Table 3, Table 4. As a whole, there were many similarities and differences between single-task and multi-task models in terms of prediction results.

Firstly, it can be seen from Table 1, Table 3 that the models built based on unpacked spinach leaves had good prediction results for both Chla. The performance of the multi-task model was slightly better than the performance of the single-task model. It can also be seen from the table that the model based on VNIR spectra performed better than the model based on NIR spectra. For the single-task model, the STBPNN model performed optimally with a *r*_*p*_ of 0.8614. For the multi-task model, the MTCNN model had the best performance with a *r*_*p*_ of 0.8394.

Secondly, Table 2, Table 4 showed satisfactory results for Chla. The table shows that the model based on VNIR spectra was superior to the model based on NIR spectra. The best model among them had *r*_*p*_ up to 0.9670. The STCNN model and the MTCNN model were slightly lower than the other two corresponding models. However, the r also exceeded 0.9 for the model based on VNIR spectra.

The results were similar for Chlb, Chlt and Car predictions, but the optimal model varied according to the composition. The overall data showed satisfactory results for both the single-task and multi-task models. The performance of the multi-task model was slightly lower than that of the single-task model. However, the multi-task model could save a lot of effort and resources compared to the single-task model, which was more suitable for practical production needs. This indicated that the assessment of vegetable freshness could be achieved more easily and quickly using the multi-task model.

### 3.2 Visualization

#### 3.2.1 Linear fitting

**Fig. 2:**
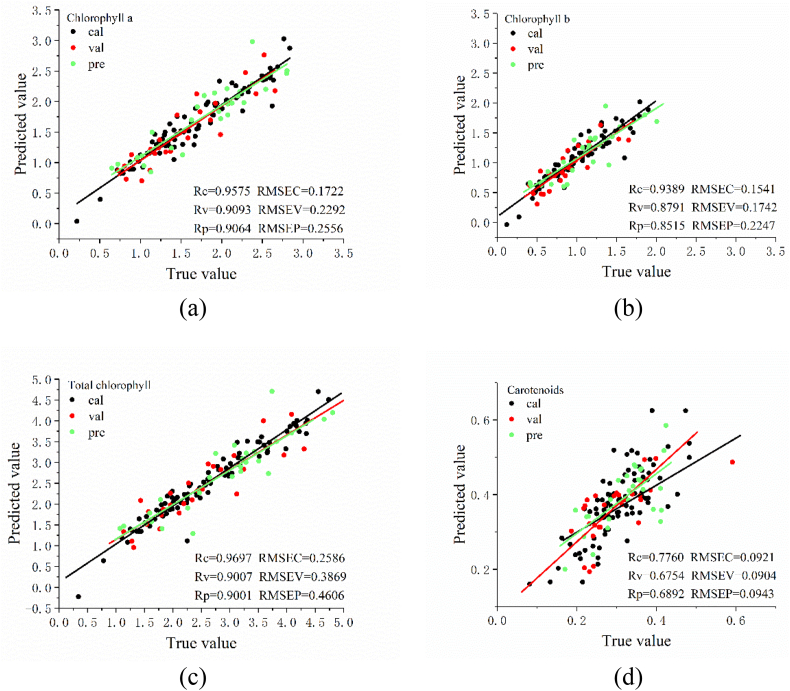
Prediction results of the single-task convolutional neural network (STCNN) model for the four metrics. (a) chlorophyll *a*; (b) chlorophyll *b*; (c) total chlorophyll; (d) carotenoids. The unit of RMSEC, RMSEV and RMSEP is mg/g.

**Fig. 3:**
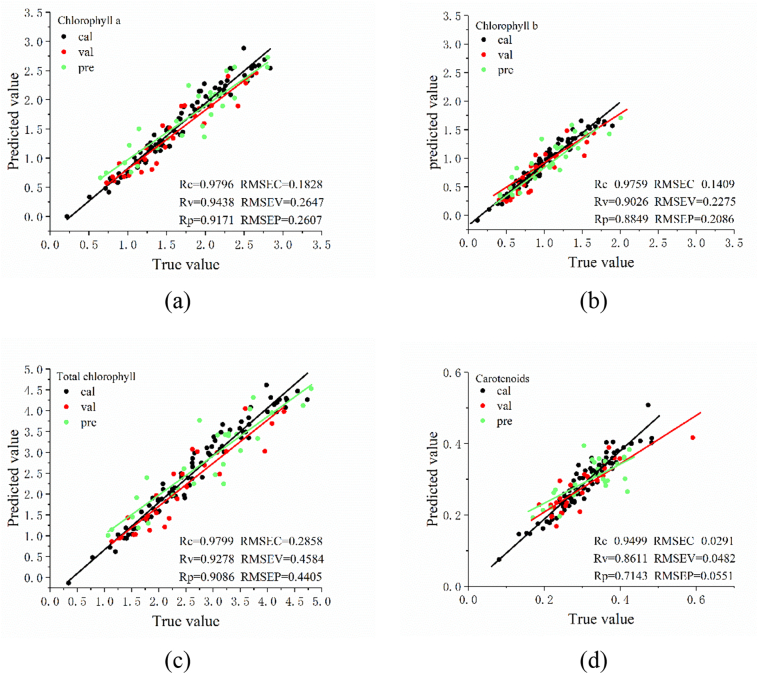
Prediction results of the multi-task convolutional neural network (MTCNN) model for the four metrics. (a) chlorophyll *a*; (b) chlorophyll *b*; (c) total chlorophyll; (d) carotenoids. The unit of RMSEC, RMSEV and RMSEP is mg/g.

To visualize the prediction results, spinach based on visible near-infrared (VNIR) spectra in the packaged state was chosen as representative data. The prediction results of the single-task and multi-task models for the training set, validation set and testing set were plotted separately, as shown in Fig. 2 and Fig. 3. From the graph, it could be intuitively seen that the closer the actual value and predicted value fit into a straight line, the better the prediction results of the model were.

Fig. 2 shows the prediction results of the single-task model for different datasets of four attributes. Fig. 2 (a) (b) (c) (d) represents the prediction results of the models for Chla, Chlb, Chlt and Car respectively. The three different colored lines in each figure represent the linear fit in different datasets, and the closer the three lines were to one line, the better the linear fit. The figure intuitively shows that Chla and Chlt were fitted relatively well, with the three fitted lines approximately overlapping completely; Car was fitted relatively poorly, with the three fitted lines relatively dispersed. This was similar to the trend of the *r* predicted by the model.

Fig. 3 shows the prediction results of the multi-task model for four attributes. Fig. 3 (a) (b) (c) (d) represents the prediction results of the model for Chla, Chlb, Chlt and Car respectively. The plots show that Chla, Chlb, and Chlt were fitted similarly to Fig. 2 (a), Fig. 2 (b) and Fig. 2 (c), which was consistent with the trend of the predicted correlation coefficients. It can also be seen that Fig. 3 (c) and Fig. 3 (d) fit better than Fig. 2 (c) and Fig. 2 (d), where the difference in the Car fitted curves can be clearly seen. This was consistent with the case of the correlation coefficients predicted by the model. It also shows that linear fitting can be used to visualize the predictions of the model.

#### 3.2.2 Important wavelengths

To be able to visualize the weights of different wavelengths for different tasks, the important wavelengths of different metrics were visualized using Grad-CAM++. STCNN and MTCNN were chosen as representative models for the visualization of Grad-CAM++, respectively. **Fig. S3**, **Fig. S4**, **Fig. S5** and **Fig. S6** show the visualization of important wavelengths of unpackaged and packaged spinach leaves models based on VNIR spectra and NIR spectra, respectively.

Comparison of **Fig. S3** (a) and **Fig. S3** (e) showed that for unpackaged spinach leaves, the STCNN model and the MTCNN model had great similarity in trend for Chla important wavelength. There was some variation between peaks and troughs. Comparing **Fig. S4** (a) and **Fig. S4** (e), there was a similar trend in the distribution of important wavelengths for both models for packaged spinach leaves. The difference was that the peak in **Fig. S4** (a) and the valley in **Fig. S4** (e) were both near 720 nm. A comparison of **Fig. S3** (a) and **Fig. S4** (a) revealed that the distribution of significant wavelengths of the two had a significant difference. Similar results would also be found by comparing **Fig. S5** (a), **Fig. S5** (e), **Fig. S6** (a), and **Fig. S6** (e) respectively.

A comparison of **Fig. S3** (b) and **Fig. S3** (e) indicated that for unpackaged spinach leaves, the two models had similarities in the distribution of the important wavelengths of Chlb. The peak of **Fig. S3** (b) and the valley of **Fig. S3** (e) were both near 717 nm, indicating that they were again somewhat different. Comparing **Fig. S4** (b) and **Fig. S4** (e), for packaged spinach leaves, the distributions of important wavelengths for both models showed a similar trend. Comparison of **Fig. S3** (b) and **Fig. S4** (b) revealed that the distribution of important wavelengths of the two had significant differences. Similar results would also be found by comparing **Fig. S5** (b), **Fig. S5** (e), **Fig. S6** (b), and **Fig. S6** (e) respectively.

A comparison of **Fig. S3** (c) and **Fig. S3** (e) showed that for unpackaged spinach leaves, the two models showed very similarity in the distribution of important wavelengths of Chlt. The wavelength distributions of its peaks and valleys almost overlapped. Comparing **Fig. S4** (c) and **Fig. S4** (e), for packaged spinach leaves, the distributions of important wavelengths for both models had similar trends. But for peak and valley wavelengths the distributions were significantly different. Comparison of **Fig. S3** (c) and **Fig. S4** (c) would show that the trends of the important wavelengths were different and the wavelength distributions of the peaks and valleys were quite different. Similar results would also be found by comparing **Fig. S5** (c), **Fig. S5** (e), **Fig. S6** (c), and **Fig. S6** (e) respectively.

A comparison of **Fig. S3** (d) and **Fig. S3** (e) showed that for unpackaged spinach leaves, the two models displayed a very similar distribution of Car important wavelengths. But the peaks and valleys of the two were very different. Comparing **Fig. S4** (d) and **Fig. S4** (e), for packaged spinach leaves, the distribution of significant wavelengths had the same trend for both models. A comparison of **Fig. S3** (d) and **Fig. S4** (d) would show that both were very similar in terms of wavelength trend and distribution of significant wavelengths, with their valleys near the wavelength 555 nm. Similar results would also be found by comparing **Fig. S5** (d), **Fig. S5** (e), **Fig. S6** (d), and **Fig. S6** (e) respectively.

The distribution of important wavelengths for the MTCNN model is shown in **Fig. S3** (e), **Fig. S4** (e), **Fig. S5** (e) and **Fig. S6** (e). By comparing **Fig. S3** (e) and **Fig. S4** (e), it could be noticed that not only the trends were extremely similar to each other, but also the peaks and valleys almost overlapped. The peaks and valleys of **Fig. S3** (e) and **Fig. S4** (e) were 673 nm, 675 nm and 717 nm, 719 nm, respectively. Likewise, a similar trend could be found by comparing **Fig. S5** (e) and **Fig. S6** (e), where the peaks and valleys were 1450 nm, 1447 nm and 1360 nm, 1356 nm, respectively.

### 3.3 Discussion

In this study, three multi-task regression models (MTPLSR, MTBPNN and MTCNN) were designed for the simultaneous prediction of Chla, Chlb, Chlt and Car of spinach stored at different periods to assess the quality of spinach based on VNIR and NIR spectra, respectively.

The usage of new modeling algorithms and consideration of samples under different storage conditions make the spinach pigment prediction model more accurate and robust.

Firstly, the utilization of deep learning methods improved the feature extraction ability and final regression performance. Among all these models, CNN based models showed relatively better performances than PLSR and BPNN models. Various studies have been reported for leaf pigment content prediction using hyperspectral imaging with deep learning models. In most studies, the CNN models have shown better or equivalent performances compared with the conventional machine learning methods (Wang, Li, Wang, & Wang, 2020, Ye et al., 2024, Zhang et al., 2023, Zhang et al., 2022). In these researches, the prediction performances of pigment content varied largely, due to the differences on the acquired samples and data. All these studies have illustrated the effectiveness of deep learning in pigment determination. However, all these studies built prediction models for each pigment.

Secondly, the presented multi-task learning strategy further improved the prediction accuracy and bring convenience for practical applications. The overall results showed that the prediction results of the multi-task models and the corresponding single-task models were close, and the performance of the single-task model might be better than that of the multi-task model. This might be because the multi-task model needed to be good for each task to get better results for prediction, so the results of each task need to be considered when partitioning the data. The single-task model was relatively flexible, there was no need to consider the correlation of the other tasks. From the above results, it could be seen that for the prediction of pigments in spinach at different periods, the *r*_*p*_ obtained by the multi-task model was similar to those obtained by the single-task model. However, the multi-task model saved a lot of time and computing resources to a certain extent for real-world applications. The attempt to use multi-task learning to predict multi-attributes have been tried in other fields, and similar results to this study were obtained (Assadzadeh, Walker, McDonald, Maharjan, & Panozzo, 2020; Cheng, Sun, Yao, Xu, & Dai, 2023).

Next, this study explored and compared the modeling performances on packaged and unpackaged samples. The results showed that different patterns were involved in these samples with different packages status, which has a certain impact on the regression accuracy. Therefore, this study took different storage conditions in to consideration and made the trained model more universal, which lacks attention in existing literature with a similar topic (Zhang et al., 2017, Vitalis, Muncan, Anantawittayanon, Kovacs, & Tsenkova, 2023). Besides, the conducted important wavelength distribution visualization revealed the similarities and the differences between the samples under different storage condition, which proved the necessity and rationality of the proposed multi-task learning algorithms (Hong et al., 2021; Sun, Cheng, Xu, & Yao, 2024).

Based on all these different results, the presented new methods obtained overall better results than the compared ones using conventional machine learning algorithms and deep learning algorithms. Zhang, Li, et al. (2023)) and Zhang, Zhang, et al. (2023)) used single-task 1DCNN for Chinese cabbage chlorophyll content inspection and realized coefficient of determination (R^2^) values of 0.52–0.64 for chlorophyll *a*, chlorophyll *b*, and total chlorophyll. Zhang et al. (2022) used single task CNN for Chinese cabbage pigments measurement (chlorophyll *a*, chlorophyll *b*, total chlorophyll and carotenoids), the R^2^ values were from 0.45 to 0.53. Wang et al. (2020) used hyperspectral imaging with different data analysis strategies to predict the chlorophyll in millet leaves, and the coefficient of determination of validation (R_V_^2^) ranging from 0.421 to 0.839, which were relatively worse than the results in this research. Ye et al. (2024) used hyperspectral imaging with different modeling methods to predict Chlt in lettuce leaves, with an average R^2^ less than 0.80. In the research of Zhang et al. (2017), hyperspectral imaging with PLSR was used to determine the pigment content in spinach leaves. The pigment content prediction for spinach leaves showed worse results for Chla, Chlb and Chlt than the present research, and the prediction results of Car were better than those in this study. The differences in these results might be attributed to acquired datasets for analysis. The model performances are highly depending on the datasets to be trained, including the spectral profiles and the content of the components to be studied. It was a fact that the samples used in these studies were different, and the sampling strategies were different. Thus, the performances varied even for the same component. Moreover, the differences of the used modeling algorithms might also affect the results.

Although impressive achievements have been made by the multi-task models, there were still limitations to be improved in future work. Wang et al. (2024) discussed the affecting factors for multi-task learning for simultaneous multi-components prediction, such as the high and low content of the components and the components with very similar chemical structures. In our research, the limitations are discussed as follows. For each sample, there were only one spectrum, and one sample had multiple quality attributes. Since there were different distribution patterns of different quality attributes, the selection of the training samples was quite important to ensure good performances for all the quality attributes. To achieve this goal, the outlier samples had to be removed first. For multi-task regression models, more restrictions should be considered than the single-task regression models. When using the established multi-task model, only one model was loaded and used to predict multiple quality attributes for unknown new samples, bringing great convenience for real-world applications. Moreover, this study used the simple multi-task regression strategy, and more multi-task regression strategies could be explored to improve the model performances.

## 4 Conclusion

In this study, hyperspectral imaging with single-task and multi-task regression models were successfully used to determine the content of Chla, Chlb, Chlt and Car in spinach leaves stored under different conditions. The results showed that the prediction performances of the multi-task models (MTPLSR, MTBPNN, and MTCNN) had similar trends to those of the corresponding single-task models (STPLSR, STBPNN, and STCNN). Although the performances of the single-task models were slightly higher than those of the multi-task model, the multi-task model not only saved a lot of energy and resources compared to the single-task model, but also was more suitable for practical production needs. Compared with the conventional machine learning models, the CNN models obtained the relatively better performances, indicating the effectiveness of CNN models. The models using spectra in the range related to pigment (VNIR) obtained relatively better performances than those using NIR spectra. This study provided a certain foundation for the multi-task modeling of pigment content in vegetables. These approaches can be extended to be used in the simultaneous determination of multiple quality attributes in various types of foods. The multi-task models have great potential to be used for real-world scenarios by building and loading only one model for multiple tasks, which overcome the shortage of single-task modeling.

## CRediT authorship contribution statement

**Mengyu He:** Writing – original draft, Validation, Software, Investigation, Data curation. **Chen Jin:** Formal analysis, Investigation. **Cheng Li:** Software, Investigation. **Zeyi Cai:** Visualization, Investigation. **Dongdong Peng:** Investigation. **Xiang Huang:** Investigation. **Jun Wang:** Investigation. **Yuanning Zhai:** Investigation. **Hengnian Qi:** Supervision, Funding acquisition. **Chu Zhang:** Writing – review & editing, Resources, Project administration, Methodology, Conceptualization.

## Declaration of competing interest

The authors declare that they have no known competing financial interests or personal relationships that could have appeared to influence the work reported in this paper.

## Data Availability

Data will be made available on request.

## Appendix A Supplementary data

Supplementary material Image 1

## References

[bb0005] Abdi H., Williams L.J. (2010). Principal component analysis. WIREs Computational Statistics.

[bb0010] Assadzadeh S., Walker C., McDonald L., Maharjan P., Panozzo J. (2020). Multi-task deep learning of near infrared spectra for improved grain quality trait predictions. Journal of Near Infrared Spectroscopy.

[bb0015] Chattopadhay A., Sarkar A., Howlader P., Balasubramanian V.N. (2018). 2018 IEEE winter conference on applications of computer vision (WACV), Lake Tahoe, NV.

[bb0020] Chen Z.Y., Wang Q.P., Zhang H., Nie P.C. (2021). Hyperspectral imaging (HSI) Technology for the non-Destructive Freshness Assessment of pearl gentian grouper under different storage conditions. Sensors.

[bb0025] Cheng J., Sun J., Yao K., Xu M., Dai C. (2023). Multi-task convolutional neural network for simultaneous monitoring of lipid and protein oxidative damage in frozen-thawed pork using hyperspectral imaging. Meat Science.

[bb0030] Cheng J.H., Sun D.W., Qu J.H., Pu H.B., Zhang X.C., Song Z.X., Zhang H. (2016). Developing a multispectral imaging for simultaneous prediction of freshness indicators during chemical spoilage of grass carp fish fillet. Journal of Food Engineering.

[bb0035] Duan H.W., Zhu R.G., Yao X.D., Lewis E. (2017). Sensitive variables extraction, non-destructive detection and visualization of total viable count (TVC) and pH in vacuum packaged lamb using hyperspectral imaging. Analytical Methods.

[bb0040] Geladi P., Kowalski B.R. (1986). Partial least-squares regression: A tutorial. Analytica Chimica Acta.

[bb0045] Gowen A.A., O’Donnell C.P., Cullen P.J., Downey G., Frias J.M. (2007). Hyperspectral imaging – An emerging process analytical tool for food quality and safety control. Trends in Food Science & Technology.

[bb0050] He J., Zhang C., Zhou L., He Y. (2021). Simultaneous determination of five micro-components in Chrysanthemum morifolium (Hangbaiju) using near-infrared hyperspectral imaging coupled with deep learning with wavelength selection. Infrared Physics & Technology.

[bb0055] Hong S.M., Baek S.S., Yun D., Kwon Y.H., Duan H.T., Pyo J., Cho K.H. (2021). Monitoring the vertical distribution of HABs using hyperspectral imagery and deep learning models. Science of the Total Environment.

[bb0060] Huang H., Zhou H., Yang X., Zhang L., Qi L., Zang A.-Y. (2019). Faster R-CNN for marine organisms detection and recognition using data augmentation. Neurocomputing.

[bb0065] Kelley S.S., Rials T.G., Snell R., Groom L.H., Sluiter A. (2004). Use of near infrared spectroscopy to measure the chemical and mechanical properties of solid wood. Wood Science and Technology.

[bb0070] Kevers C., Falkowski M., Tabart J., Defraigne J.-O., Dommes J., Pincemail J. (2007). Evolution of antioxidant capacity during storage of selected fruits and vegetables. Journal of Agricultural and Food Chemistry.

[bb0075] Kidmose U., Edelenbos M., Christensen L.P., Hegelund E. (2005). Chromatographic determination of changes in pigments in spinach (*Spinacia oleracea* L.) during processing. Journal of Chromatographic Science.

[bb0080] Kumar B.R. (2017). Application of HPLC and ESI-MS techniques in the analysis of phenolic acids and flavonoids from green leafy vegetables (GLVs). Journal of Pharmaceutical Analysis.

[bb0085] Li H., Ren H., Liu Z., Huang F., Xia G., Long Y. (2022). In-situ monitoring system for weld geometry of laser welding based on multi-task convolutional neural network model. Measurement.

[bb0090] Li J., Cheng J.-H., Shi J.-Y., Huang F. (2012).

[bb0095] Li Y., Zheng X., Zhang D., Li X., Fang F., Chen L. (2021). Rapid nondestructive simultaneous detection for physicochemical properties of different types of sheep meat cut using portable Vis/NIR reflectance spectroscopy system. Foods.

[bb0100] Limantara L., Dettling M., Indrawati R., Indriatmoko, Brotosudarmo T.H.P. (2015). Analysis on the chlorophyll content of commercial green leafy vegetables. Procedia Chemistry.

[bb0105] Liu Q., Chen S.X., Zhou D.D., Ding C., Wang J.H., Zhou H.S., Li P.X. (2021). Nondestructive detection of weight loss rate, surface color, vitamin C content, and firmness in Mini-Chinese cabbage with Nanopackaging by Fourier transform-near infrared spectroscopy. Foods.

[bb0110] Liu Y., Wang X., Tan T., Ma X., Xu L. (2020). Prediction of direct coal liquefaction residue catalytic gasification based on back propagation neural network. Energy Sources, Part A: Recovery, Utilization, and Environmental Effects.

[bb0115] Malek S., Melgani F., Bazi Y. (2018). One-dimensional convolutional neural networks for spectroscopic signal regression. Journal of Chemometrics.

[bb0120] Meghar K., Tran T., Delgado L.F., Ospina M.A., Moreno J.L., Luna J., Davrieux F. (2023). Hyperspectral imaging for the determination of relevant cooking quality traits of boiled cassava. Journal of the Science of Food and Agriculture.

[bb0125] Mishra P., Passos D. (2022). Multi-output 1-dimensional convolutional neural networks for simultaneous prediction of different traits of fruit based on near-infrared spectroscopy. Postharvest Biology and Technology.

[bb0130] Mishra P., Verschoor J., Vries M.N.-D., Polder G., Boer M.P. (2023). Portable near-infrared spectral imaging combining deep learning and chemometrics for dry matter and soluble solids prediction in intact kiwifruit. Infrared Physics & Technology.

[bb0135] Qin J., Zhang Y., Fan S., Hu X., Huang Y., Lu Z., Liu Y. (2022). Multi-task short-term reactive and active load forecasting method based on attention-LSTM model. International Journal of Electrical Power & Energy Systems.

[bb0140] Salehi F. (2020). Recent advances in the modeling and predicting quality parameters of fruits and vegetables during postharvest storage: A review. International Journal of Fruit Science.

[bb0145] Siche R., Vejarano R., Aredo V., Velasquez L., Saldaña E., Quevedo R. (2016). Evaluation of food quality and safety with hyperspectral imaging (HSI). Food Engineering Reviews.

[bb0150] Siripongvutikorn S., Usawakesmanee W., Pisuchpen S., Khatcharin N., Rujirapong C. (2023). Quality changes during storage in Thai indigenous leafy vegetable, Liang leaves (*Gnetum gnemon* var. tenerum) after different preparation methods. Italian Journal of Food Science.

[bb0155] Song Y., Cao S., Chu X., Zhou Y., Xu Y., Sun T., Zhou G., Liu X. (2023). Non-destructive detection of moisture and fatty acid content in rice using hyperspectral imaging and chemometrics. Journal of Food Composition and Analysis.

[bb0160] Spinardi A., Cocetta G., Baldassarre V., Ferrante A., Mignani I. (2010). Quality changes during storage of spinach and lettuce baby leaf. Acta Horticulturae.

[bb0165] Squeo G., De Angelis D., Summo C., Pasqualone A., Caponio F., Amigo J.M. (2022). Assessment of macronutrients and alpha-galactosides of texturized vegetable proteins by near infrared hyperspectral imaging. Journal of Food Composition and Analysis.

[bb0170] Sun J., Cheng J.H., Xu M., Yao K.S. (2024). A method for freshness detection of pork using two-dimensional correlation spectroscopy images combined with dual-branch deep learning. Journal of Food Composition and Analysis.

[bb0175] Thung K.-H., Wee C.-Y. (2018). A brief review on multi-task learning. Multimedia Tools and Applications.

[bb0180] Vitalis F., Muncan J., Anantawittayanon S., Kovacs Z., Tsenkova R. (2023). Aquaphotomics monitoring of lettuce freshness during cold storage. Foods.

[bb0185] Wang F.Y., Lin H., Xu P.T., Bi X.K., Sun L. (2021). Egg freshness evaluation using transmission and reflection of NIR spectroscopy coupled multivariate analysis. FOODS.

[bb0190] Wang X.Y., Li Z.W.L., Wang W.J., Wang J.W. (2020). Chlorophyll content for millet leaf using hyperspectral imaging and an attention-convolutional neural network. Ciência Rural.

[bb0195] Wang Y.Y., Wang S.M., Yuan Y.W., Li X.Y., Bai R.B., Wan X.F., Nan T.G., Yang J., Huang L.Q. (2024). Fast prediction of diverse rare ginsenoside contents in Panax ginseng through hyperspectral imaging assisted with the temporal convolutional network-attention mechanism (TCNA) deep learning. Food Control.

[bb0200] Wieme J., Mollazade K., Malounas I., Zude-Sasse M., Zhao M., Gowen A., Argyropoulos D., Fountas S., Van Beek J. (2022). Application of hyperspectral imaging systems and artificial intelligence for quality assessment of fruit, vegetables and mushrooms: A review. Biosystems Engineering.

[bb0205] Worsham J., Kalita J. (2020). Multi-task learning for natural language processing in the 2020s: Where are we going?. Pattern Recognition Letters.

[bb0210] Ye Z., Tan X., Dai M., Chen X., Zhong Y., Zhang Y., Ruan Y., Kong D. (2024). A hyperspectral deep learning attention model for predicting lettuce chlorophyll content. Plant Methods.

[bb0215] Zhang C., Li C., He M., Cai Z., Feng Z., Qi H., Zhou L. (2023). Leaf water content determination of oilseed rape using near-infrared hyperspectral imaging with deep learning regression methods. Infrared Physics & Technology.

[bb0220] Zhang C., Wang Q., Liu F., He Y., Xiao Y. (2017). Rapid and non-destructive measurement of spinach pigments content during storage using hyperspectral imaging with chemometrics. Measurement.

[bb0225] Zhang C., Wu W., Zhou L., Cheng H., Ye X., He Y. (2020). Developing deep learning based regression approaches for determination of chemical compositions in dry black goji berries (*Lycium ruthenicum* Murr.) using near-infrared hyperspectral imaging. Food Chemistry.

[bb0230] Zhang D., Zhang J., Peng B., Wu T., Jiao Z., Lu Y., Li G., Fan X., Shen S., Gu A. (2023). Hyperspectral model based on genetic algorithm and SA-1DCNN for predicting Chinese cabbage chlorophyll content. Scientia Horticulturae.

[bb0235] Zhang J., Zhang D., Cai Z., Wang L., Wang J., Sun L., Fan X., Shen S., Zhao J. (2022). Spectral technology and multispectral imaging for estimating the photosynthetic pigments and SPAD of the Chinese cabbage based on machine learning. Computers and Electronics in Agriculture.

[bb0240] Zhu S.S., Feng L., Zhang C., Bao Y.D., He Y. (2019). Identifying freshness of spinach leaves stored at different temperatures using hyperspectral imaging. FOODS.

[bb0245] Zhuang Q.B., Peng Y.K., Yang D.Y., Wang Y.L., Zhao R.H., Chao K.L., Guo Q.H. (2022). Detection of frozen pork freshness by fluorescence hyperspectral image. Journal of Food Engineering.

