# Supplementary material for: Simultaneous determination of pigments of spinach (Spinacia oleracea L.) leaf for quality inspection using hyperspectral imaging and multi-task deep learning regression approaches
Source: Food Chem X. 2024 May 17;22:101481. doi: 10.1016/j.fochx.2024.101481 (PMC11152701; doi:10.1016/j.fochx.2024.101481)
Supplement: Supplementary file 1 — Supplementary material [file mmc1.docx]

**Supplementary Materials**

**Figure Captions**

**Figure S1** STCNN architectures. (a) STCNN model constructed based on visible NIR spectra; (b) STCNN model built based on VIR spectra.

**Figure S2** MTCNN architecture.

**Figure S3** Visualization of important wavelengths based on VNIR (FX10) spectra for unpackaged spinach leaf prediction model. (a) (b) (c) (d) are visualizations of chlorophyll a, chlorophyll b, total chlorophyll and carotenoid important wavelengths by single-task models, respectively. (e) is the visualization of the multi-task model for chlorophyll a, chlorophyll b, total chlorophyll and carotenoid important wavelengths simultaneously.

**Figure S4** Visualization of important wavelengths in a packaged spinach leaf prediction model based on VNIR (FX10) spectra. (a) (b) (c) (d) are visualizations of chlorophyll a, chlorophyll b, total chlorophyll and carotenoid important wavelengths by single-task models, respectively. (e) is the visualization of the multi-task model for chlorophyll a, chlorophyll b, total chlorophyll and carotenoid important wavelengths simultaneously.

**Figure S5** Visualization of important wavelengths based on NIR (FX17) spectra for unpackaged spinach leaf prediction model. (a) (b) (c) (d) are visualizations of chlorophyll a, chlorophyll b, total chlorophyll and carotenoid important wavelengths by single-task models, respectively. (e) is the visualization of the multi-task model for chlorophyll a, chlorophyll b, total chlorophyll and carotenoid important wavelengths simultaneously.

**Figure S6** Visualization of important wavelengths in a packaged spinach leaf prediction model based on NIR (FX17) spectra. (a) (b) (c) (d) are visualizations of chlorophyll a, chlorophyll b, total chlorophyll and carotenoid important wavelengths by single-task models, respectively. (e) is the visualization of the multi-task model for chlorophyll a, chlorophyll b, total chlorophyll and carotenoid important wavelengths simultaneously.

| 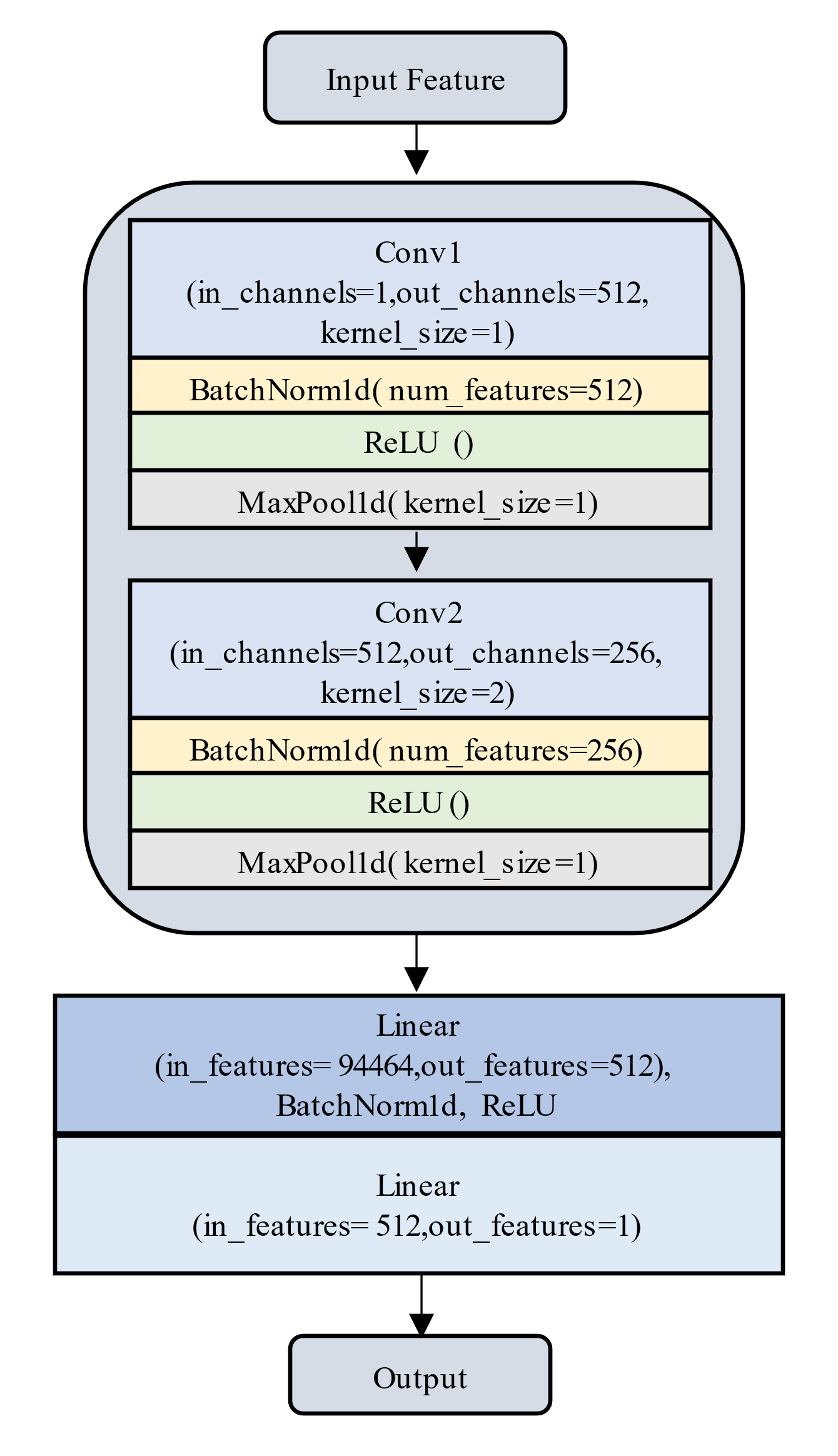 | 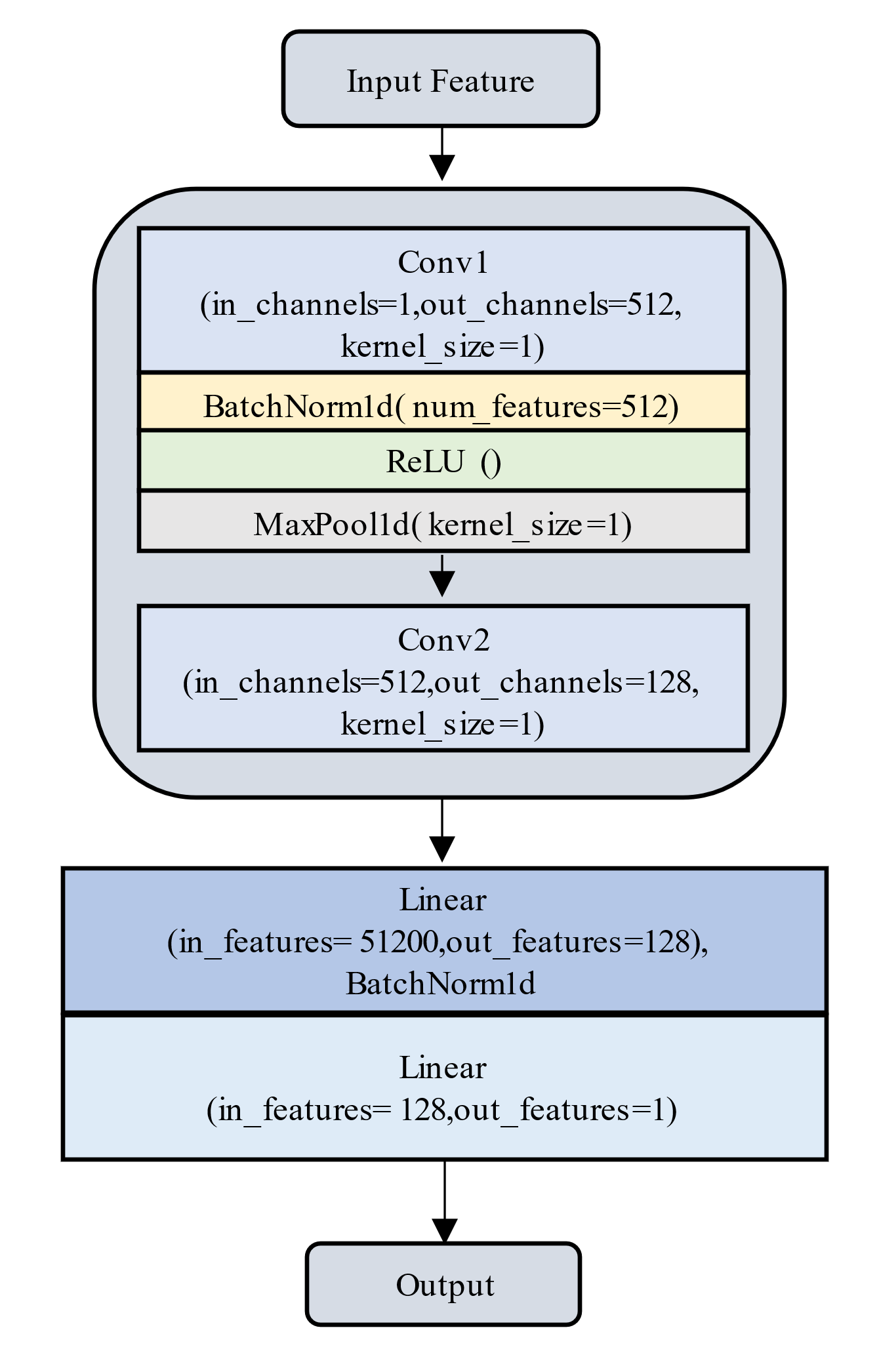 |
| --- | --- |
| (a) | (b) |
| **Figure S1** STCNN architectures. (a) STCNN model constructed based on visible NIR spectra; (b) STCNN model built based on VIR spectra.   \| 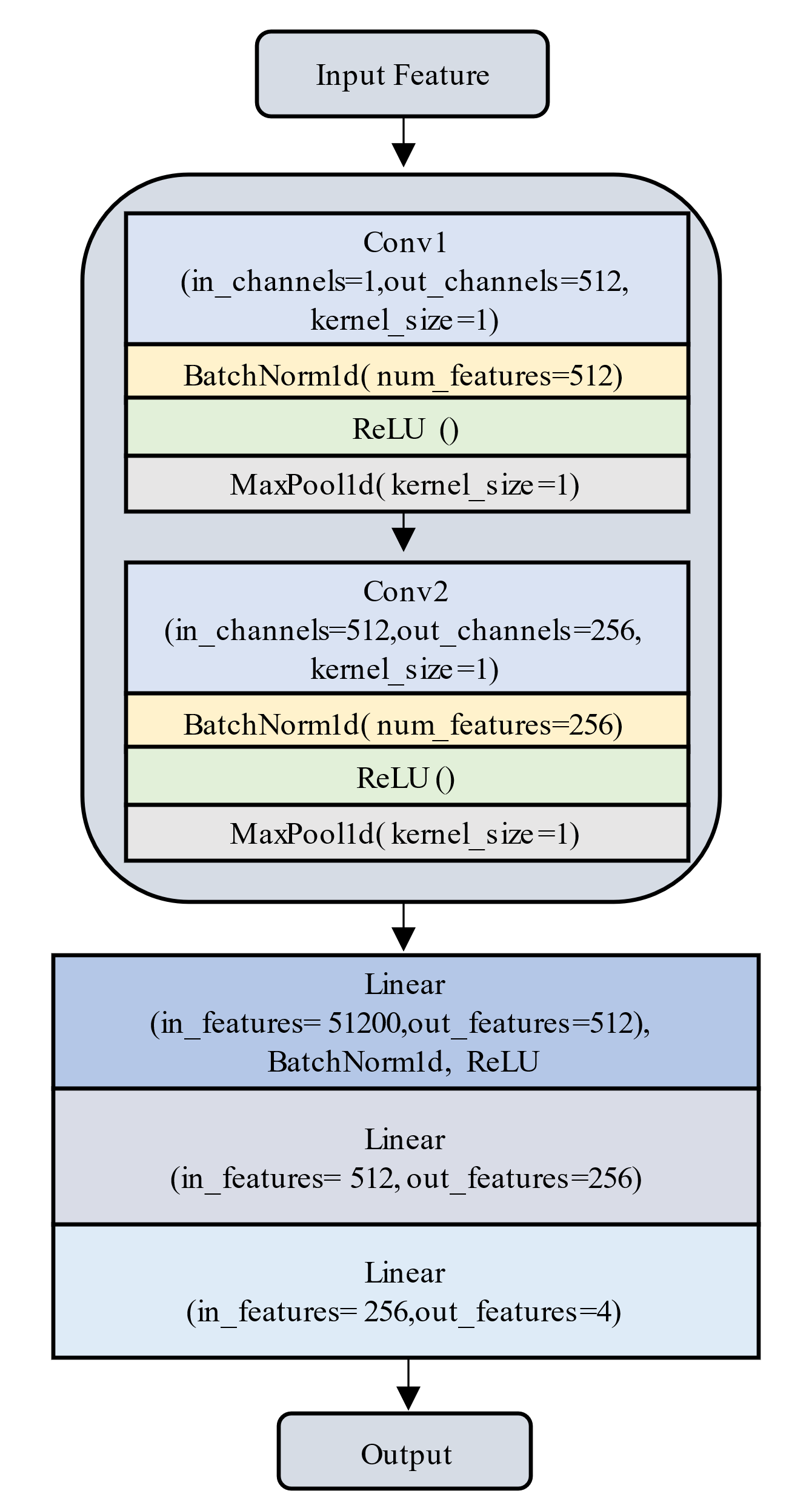 \| \| --- \| \| **Figure S2** MTCNN architecture. \| | |
| 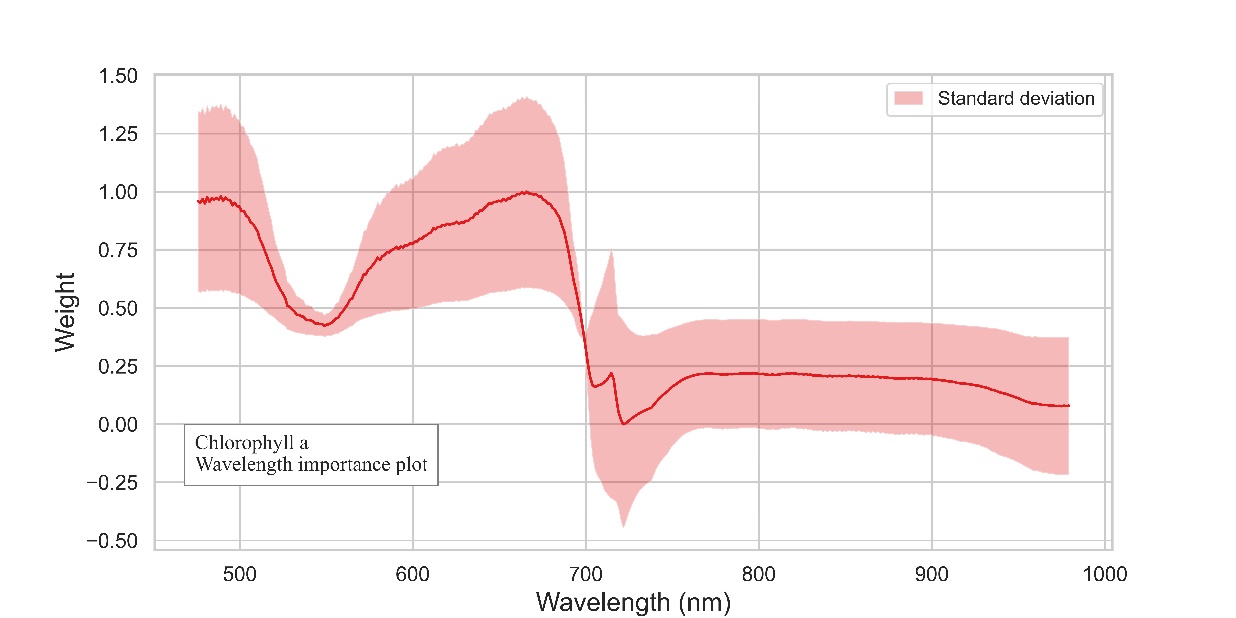 | |
| (a) | |
| 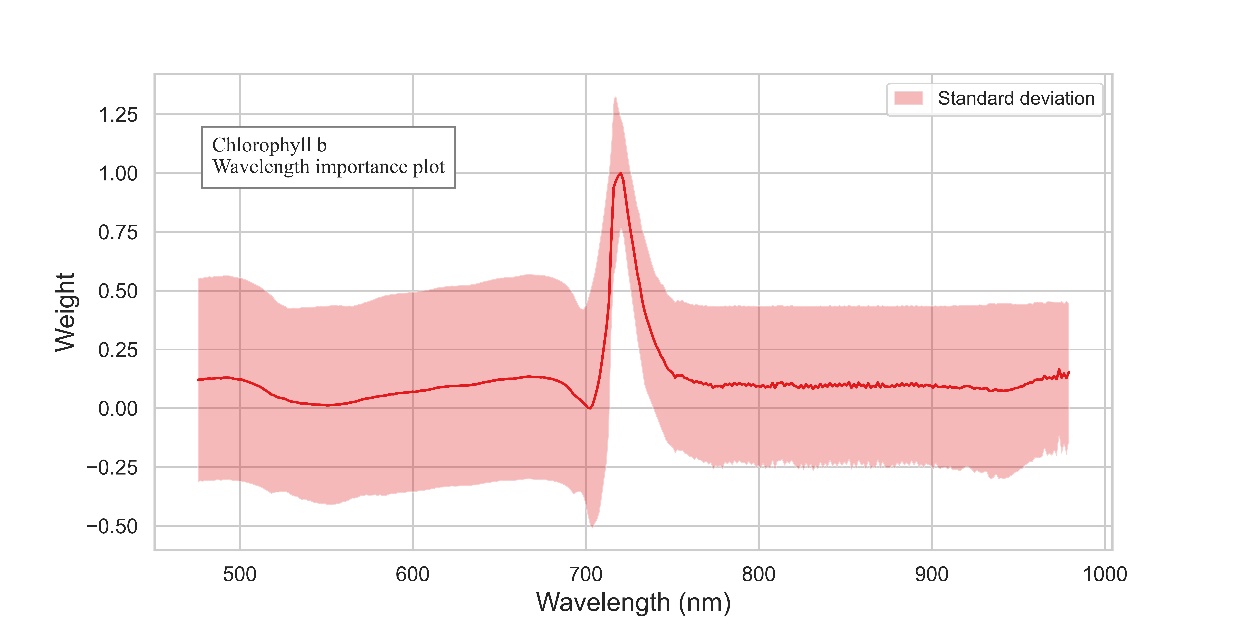 | |
| (b) | |
| 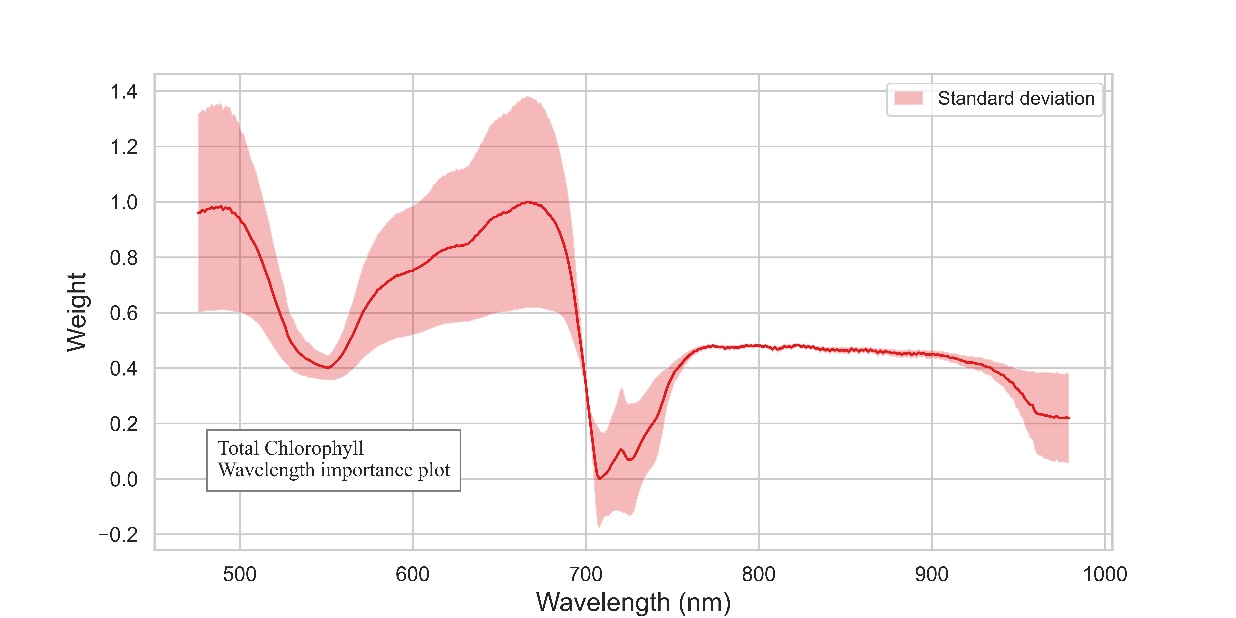 | |
| (c) | |
| 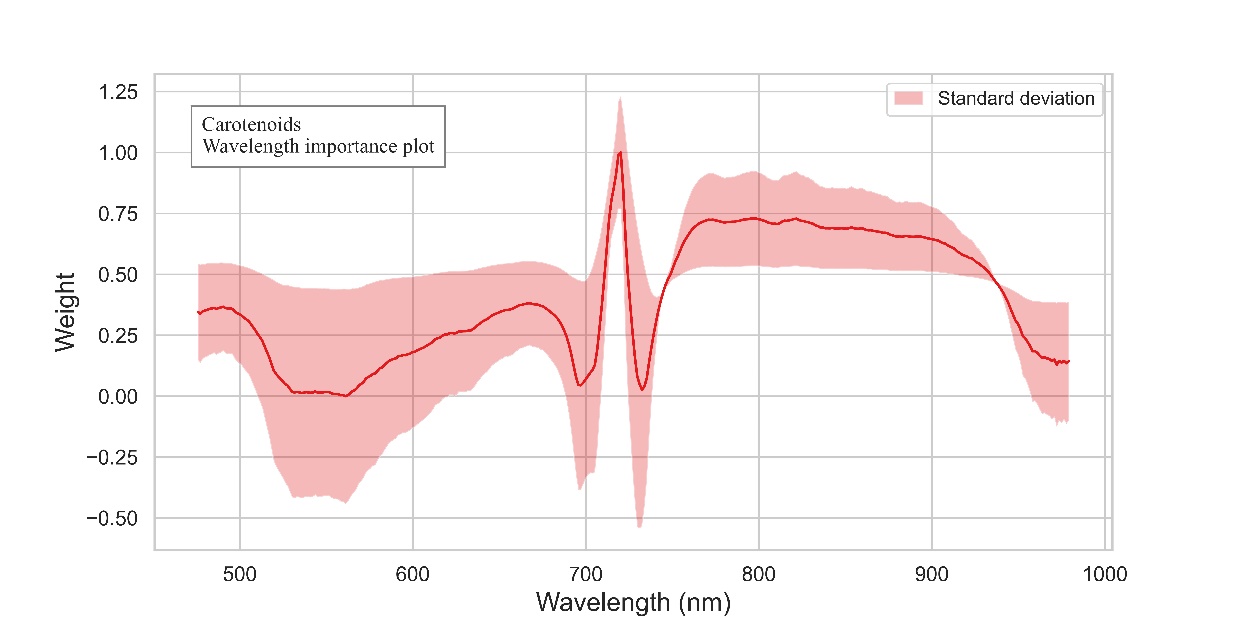 | |
| (d) | |
| 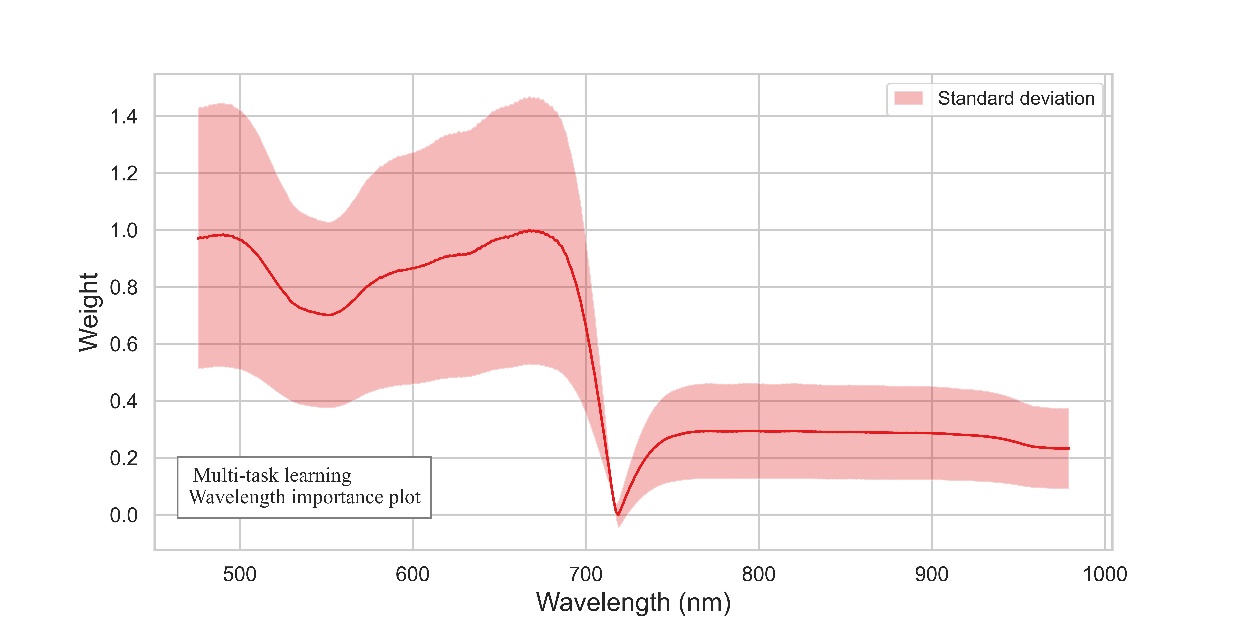 | |
| (e) | |

**Figure S3** Visualization of significant wavelengths based on VNIR (FX10) spectra for unpackaged spinach leaf prediction model. Where (a) (b) (c) (d) are visualizations of chlorophyll a, chlorophyll b, total chlorophyll and carotenoid important wavelengths by single-task models, respectively. (e) is the visualization of the multi-task model for chlorophyll a, chlorophyll b, total chlorophyll and carotenoid important wavelengths simultaneously. The unit of the y axis is a.u.

| 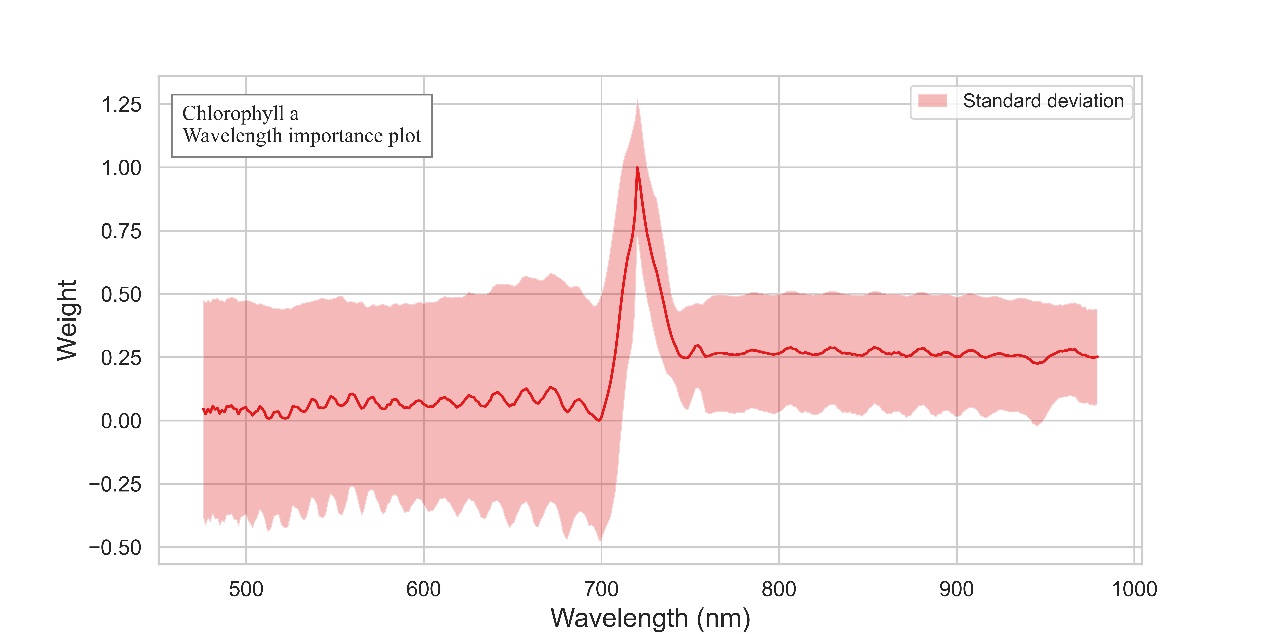 |
| --- |
| (a) |
| 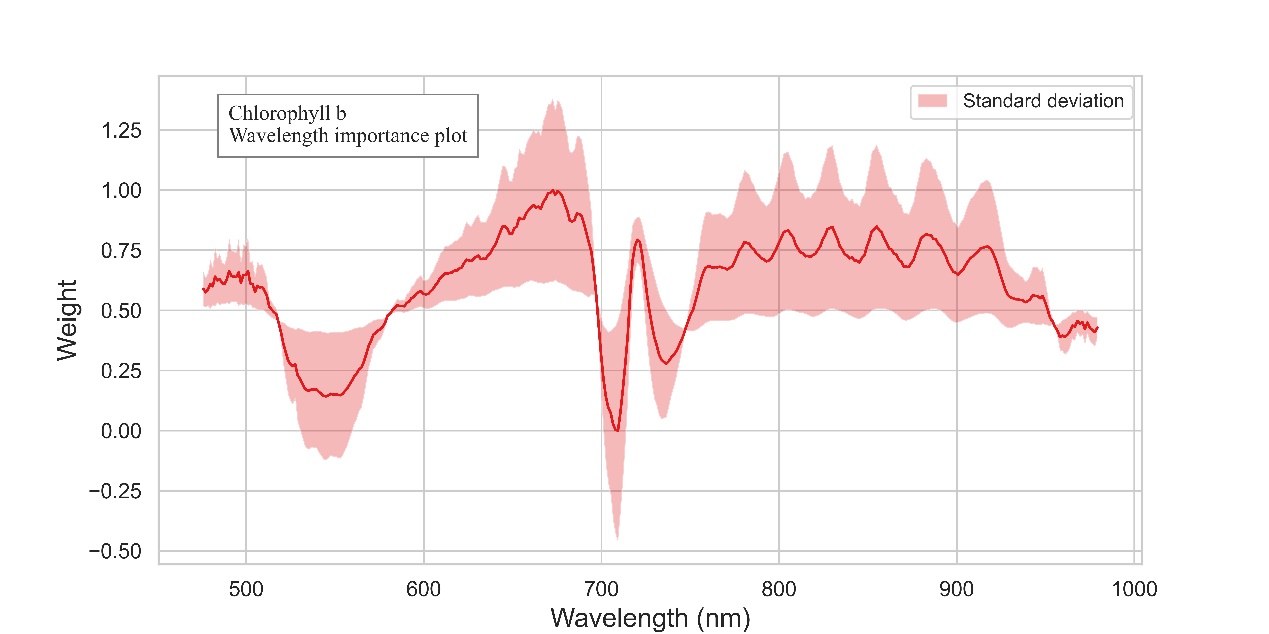 |
| (b) |
| 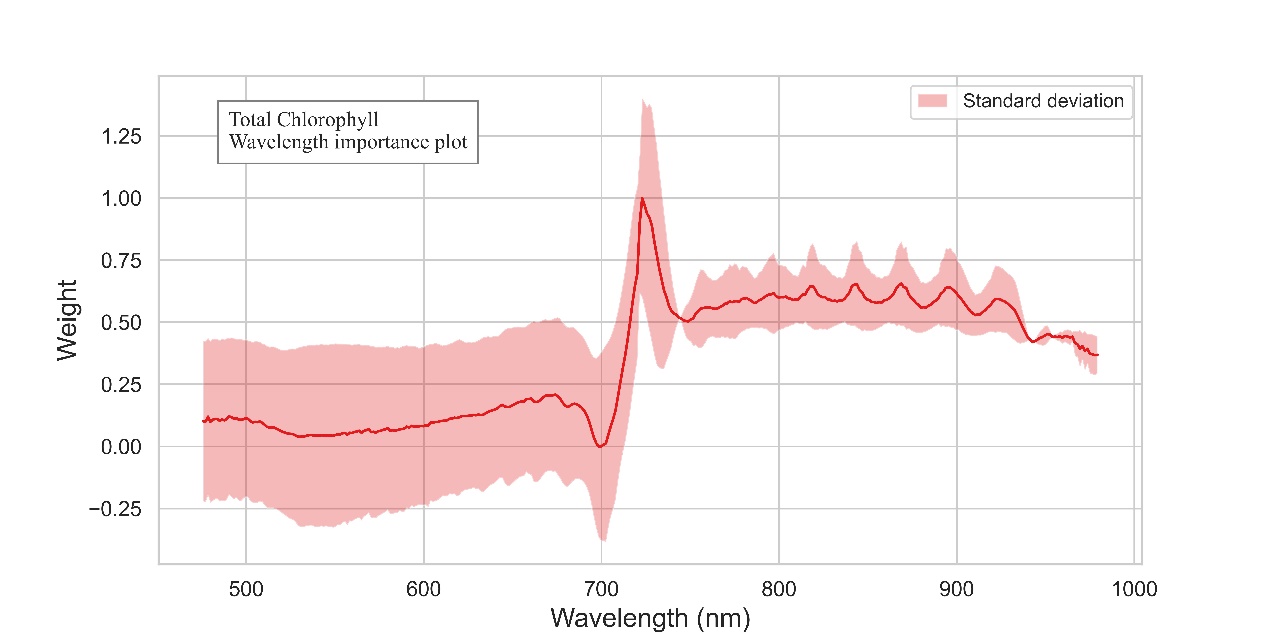 |
| (c) |
| 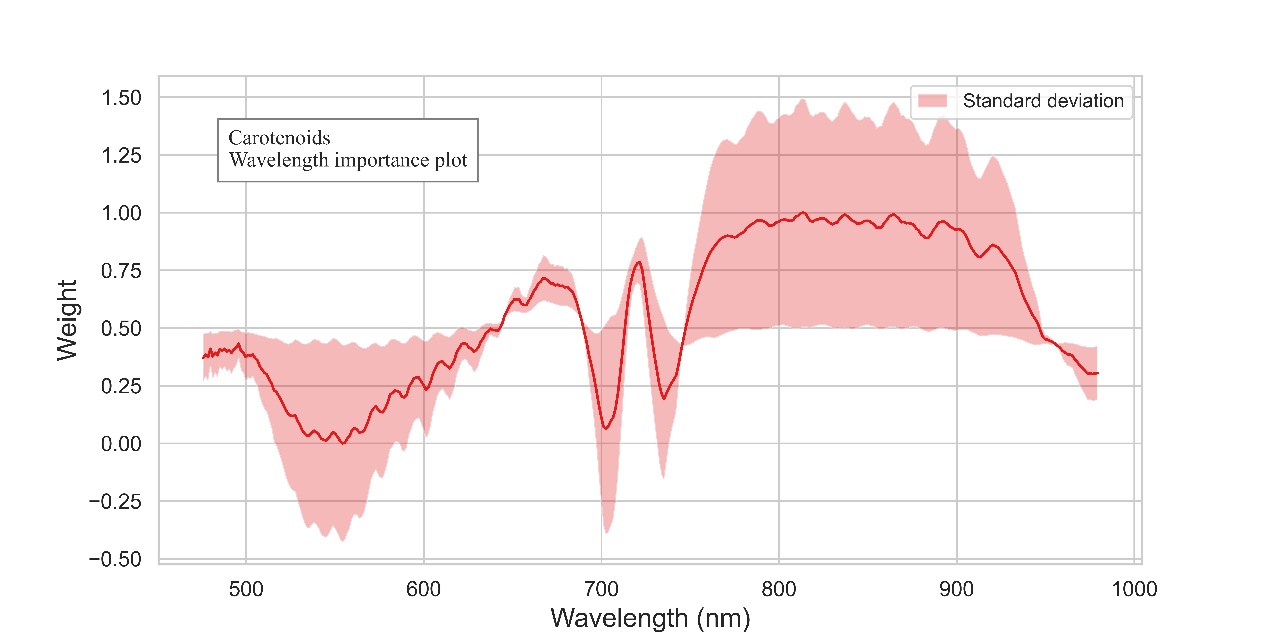 |
| (d) |
| 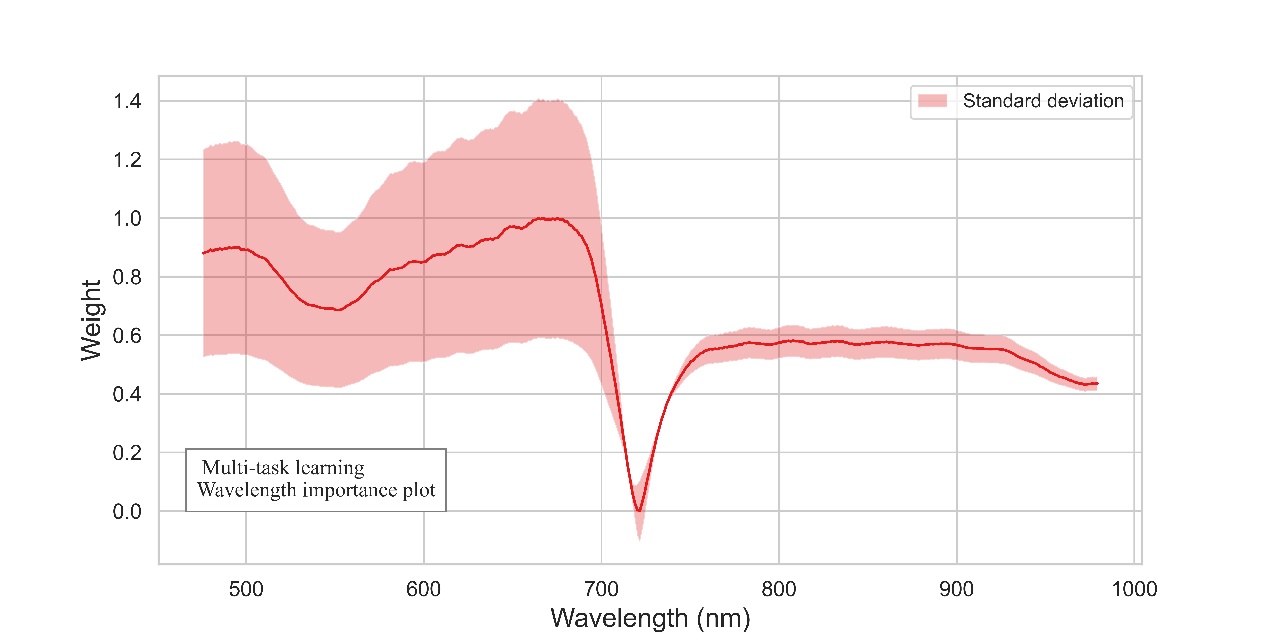 |
| (e) |

**Figure S4** Visualization of important wavelengths for packaged spinach leaf prediction model based on VNIR (FX10) spectroscopy. Where (a) (b) (c) (d) are visualizations of chlorophyll a, chlorophyll b, total chlorophyll and carotenoid important wavelengths by single-task models, respectively. (e) is the visualization of the multi-task model for chlorophyll a, chlorophyll b, total chlorophyll and carotenoid important wavelengths simultaneously. The unit of the y axis is a.u.

| 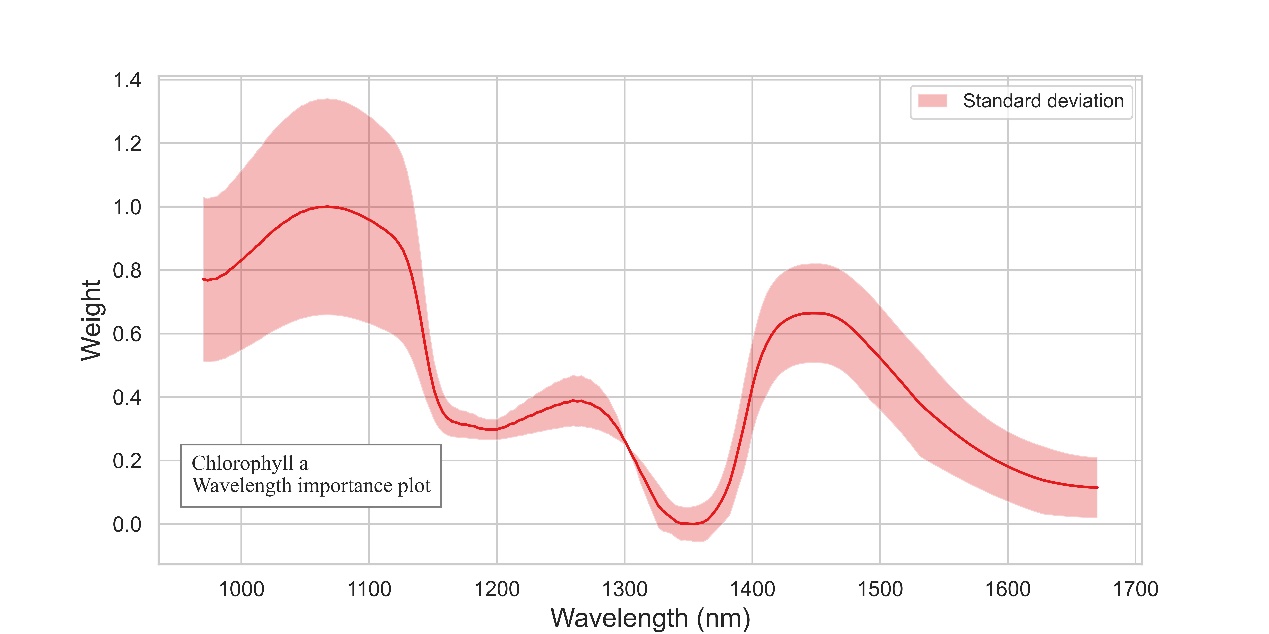 |
| --- |
| (a) |
| 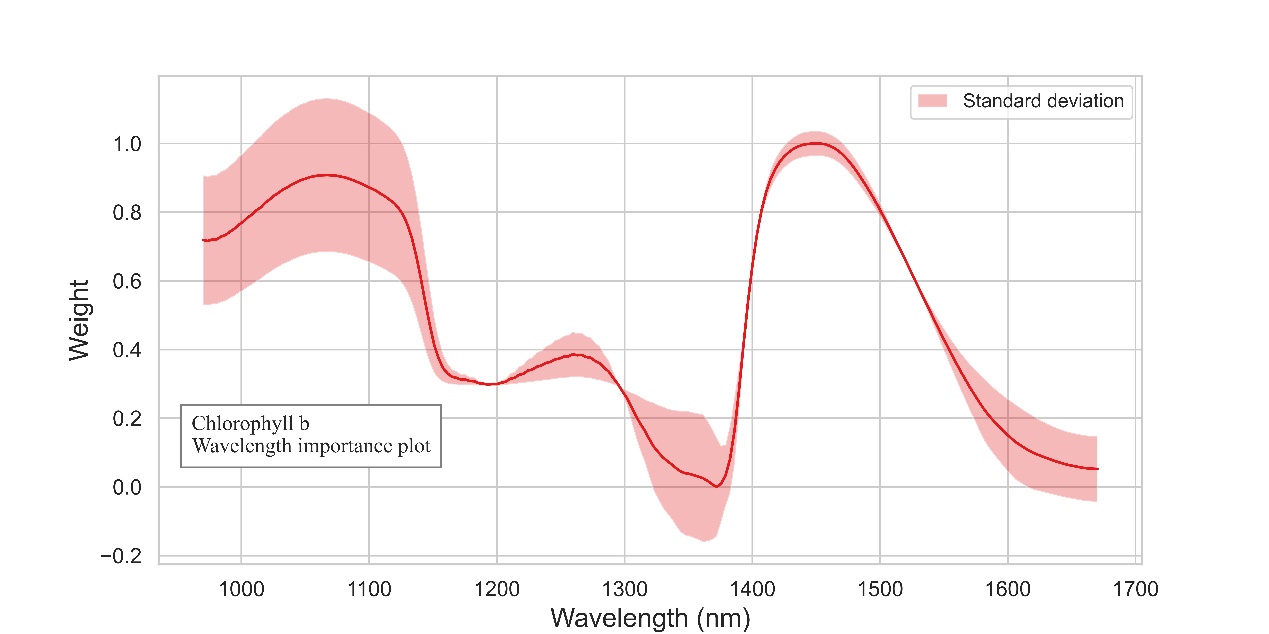 |
| (b) |
| 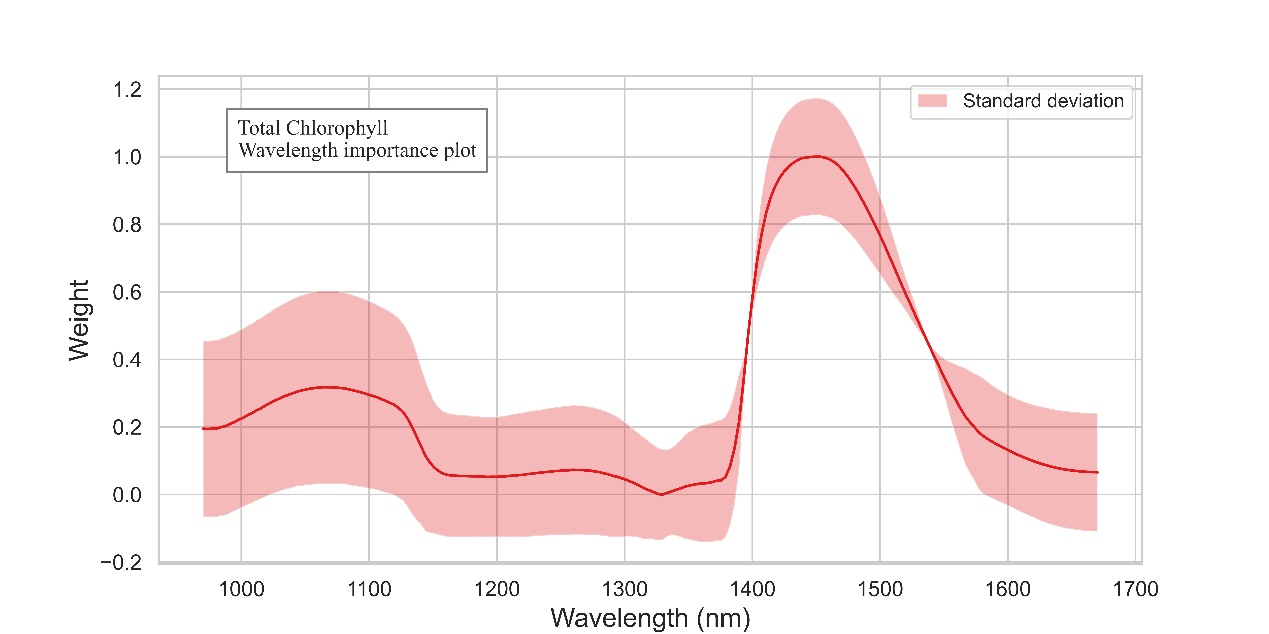 |
| (c) |
| 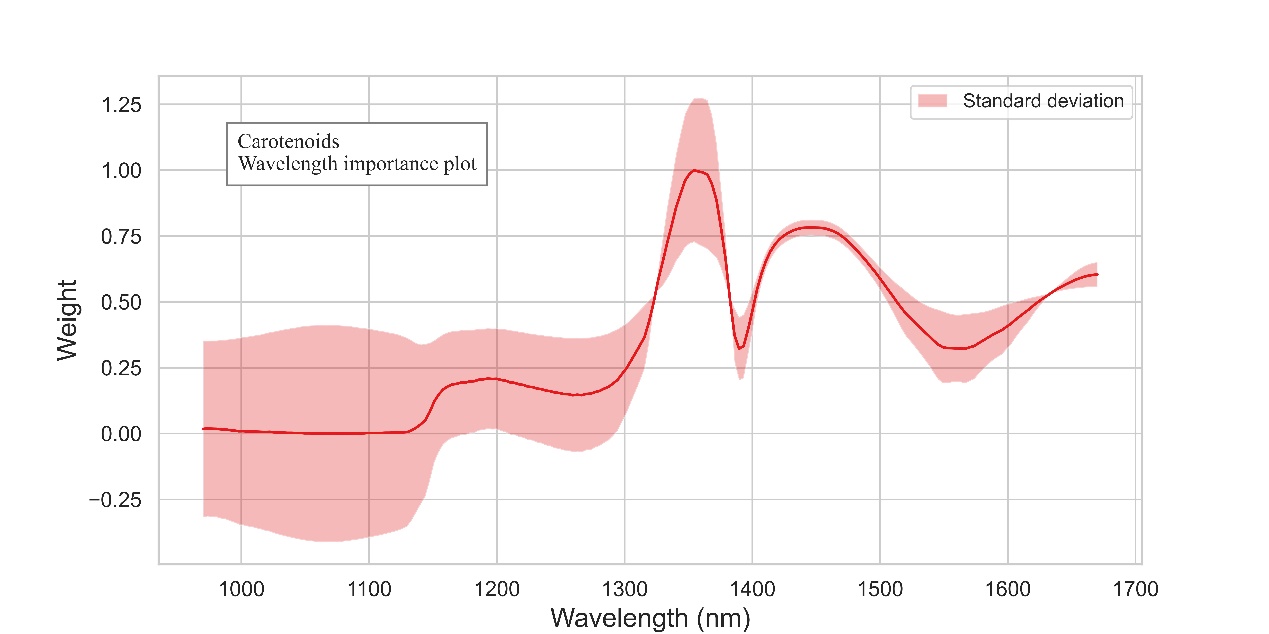 |
| (d) |
| 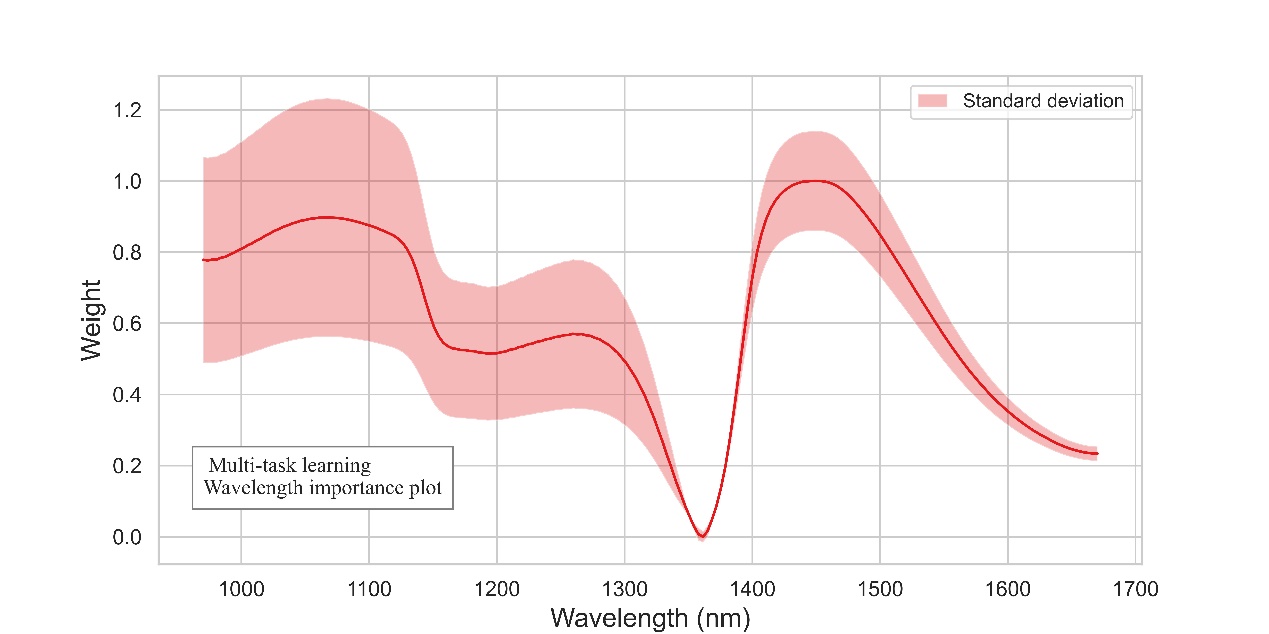 |
| (e) |

**Figure S5** Visualization of significant wavelengths based on NIR (FX17) spectra for unpackaged spinach leaf prediction model. Where (a) (b) (c) (d) are visualizations of chlorophyll a, chlorophyll b, total chlorophyll and carotenoid important wavelengths by single-task models, respectively. (e) is the visualization of the multi-task model for chlorophyll a, chlorophyll b, total chlorophyll and carotenoid important wavelengths simultaneously. The unit of the y axis is a.u.

| 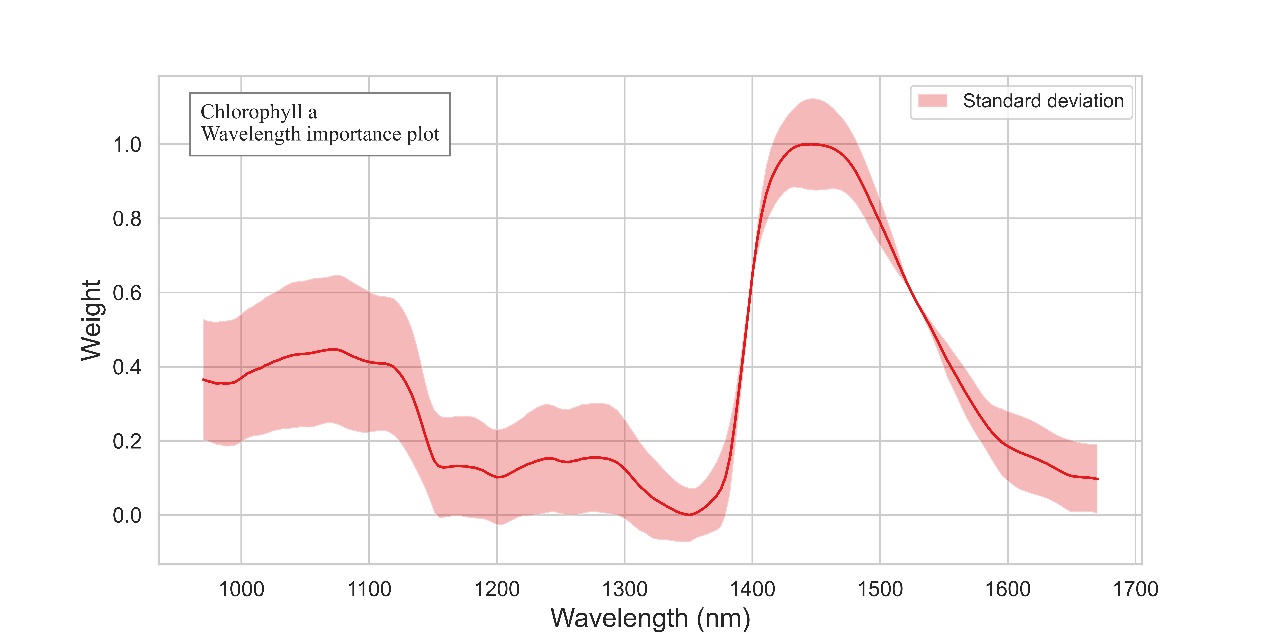 |
| --- |
| (a) |
| 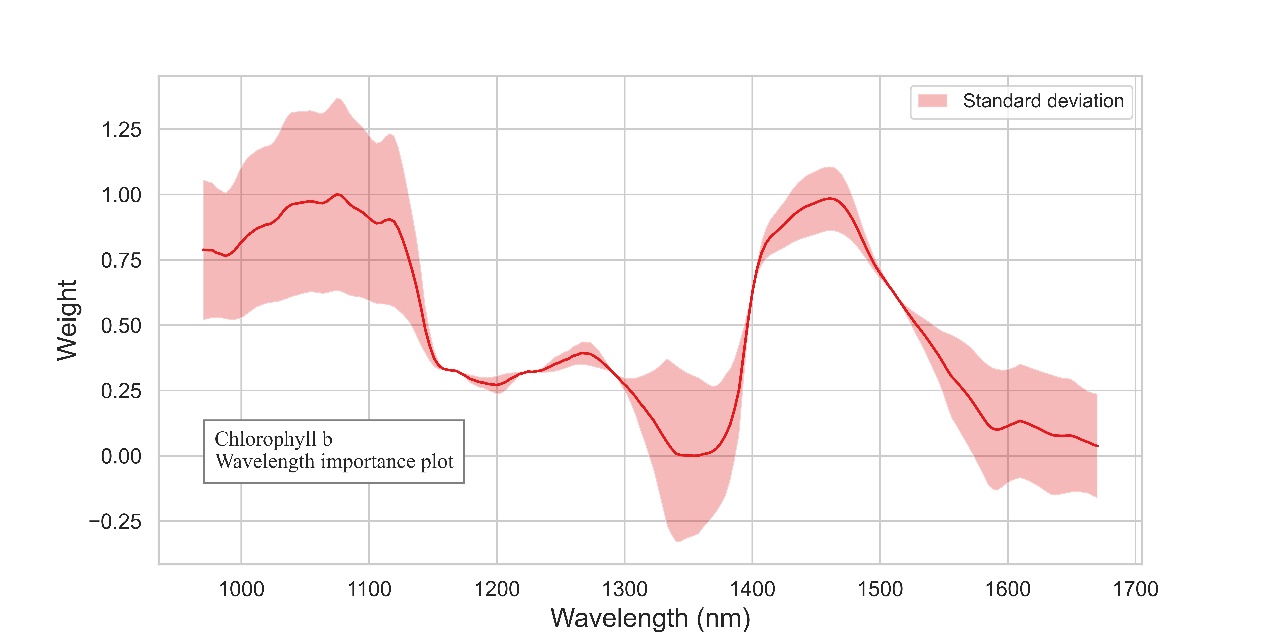 |
| (b) |
| 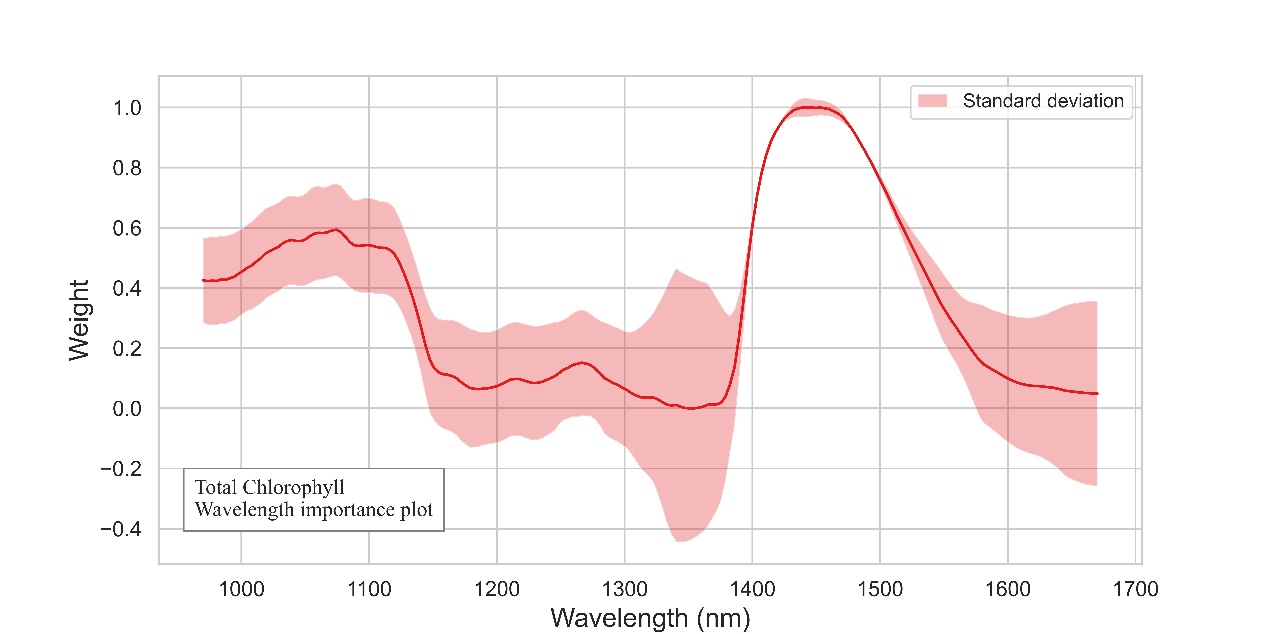 |
| (c) |
| 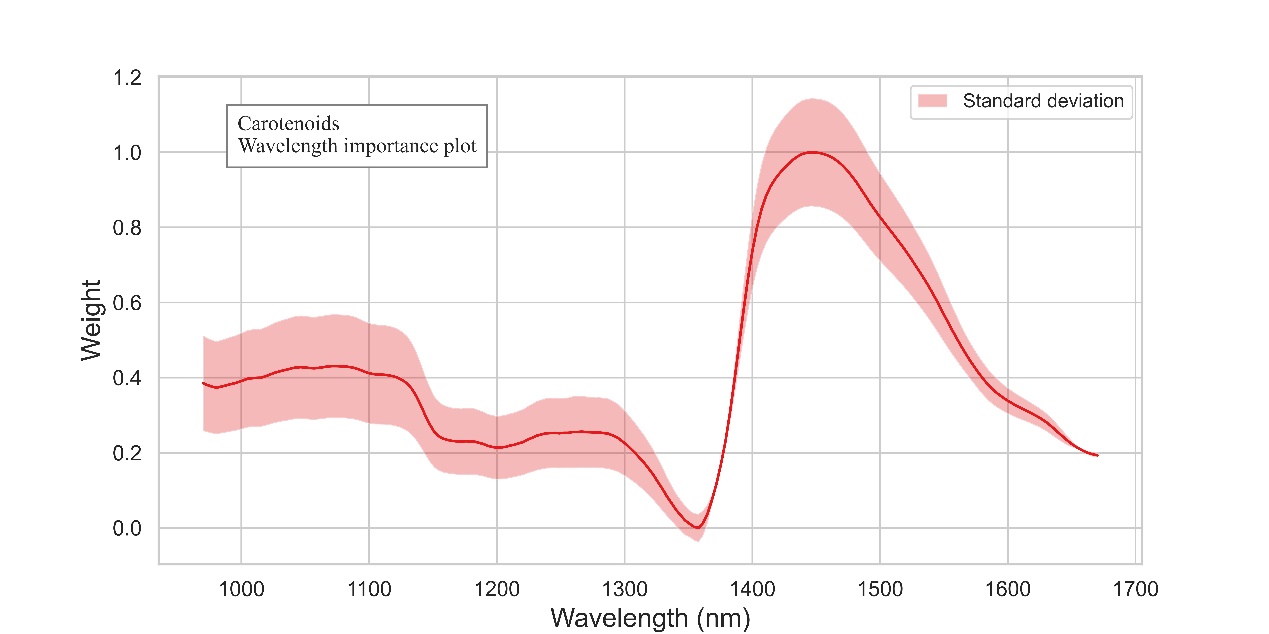 |
| (d) |
| 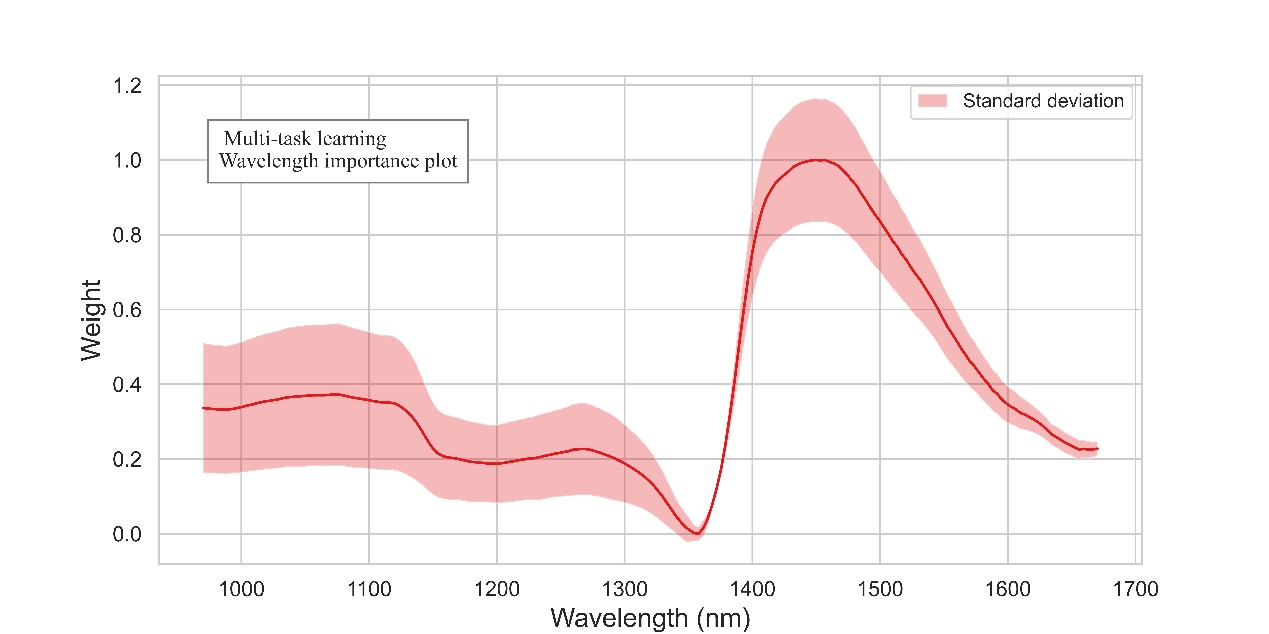 |
| (e) |

**Figure S6** Visualization of important wavelengths for packaged spinach leaf prediction model based on NIR (FX17) spectra. Where (a) (b) (c) (d) are visualizations of chlorophyll a, chlorophyll b, total chlorophyll and carotenoid important wavelengths by single-task models, respectively. (e) is the visualization of the multi-task model for chlorophyll a, chlorophyll b, total chlorophyll and carotenoid important wavelengths simultaneously. The unit of the y axis is a.u.

**Table S1** The split of training, validation and testing sets and the statistical analysis of pigments content for VNIR spectra. (Chla represents the chlorophyll a, Chlb represents the chlorophyll b, Chlt represents the total chlorophyll, and Car represents the carotenoids.)

| Datasets | Status | Number | Content Range (mg/g) | | | |
| --- | --- | --- | --- | --- | --- | --- |
|  |  |  | Chla | Chlb | Chlt | Car |
| Training | unpackaged | 61 | 1.1-2.3 | 0.5-1.5 | 1.6-3.8 | 0.2-0.4 |
|  | packaged | 84 | 0.2-2.8 | 0.1-1.9 | 0.3-4.7 | 0.1-0.5 |
| Validation | unpackaged | 19 | 1.1-2.7 | 0.6-1.5 | 1.6-4.2 | 0.2-0.4 |
|  | packaged | 27 | 0.7-2.7 | 0.4-1.7 | 1.1-4.3 | 0.2-0.6 |
| Testing | unpackaged | 19 | 1.2-2.6 | 0.7-1.5 | 1.9-3.9 | 0.2-0.4 |
|  | packaged | 27 | 0.6-2.8 | 0.4-2.0 | 1.1-4.8 | 0.2-0.4 |

**Table S2** The split of training, validation and testing sets and the statistical analysis of pigments content for NIR spectra. (Chla represents the chlorophyll a, Chlb represents the chlorophyll b, Chlt represents the total chlorophyll, and Car represents the carotenoids.)

| Datasets | Status | Number | Content Range (mg/g) | | | |
| --- | --- | --- | --- | --- | --- | --- |
|  |  |  | Chla | Chlb | Chlt | Car |
| Training | unpackaged | 58 | 1.0-2.7 | 0.5-1.6 | 1.5-4.2 | 0.2-0.4 |
|  | packaged | 79 | 0.6-3.1 | 0.3-1.7 | 1.0-4.9 | 0.2-0.6 |
| Validation | unpackaged | 19 | 1.2-2.6 | 0.7-1.5 | 1.9-4.0 | 0.2-0.4 |
|  | packaged | 25 | 0.2-2.7 | 0.1-1.7 | 0.3-4.4 | 0.1-0.4 |
| Testing | unpackaged | 19 | 1.2-2.3 | 0.6-1.5 | 1.8-3.8 | 0.2-0.4 |
|  | packaged | 25 | 0.8-2.8 | 0.5-1.9 | 1.3-4.7 | 0.2-0.4 |
